# Proteomic and transcriptomic study of brain microvessels in neonatal and adult mice

**DOI:** 10.1371/journal.pone.0171048

**Published:** 2017-01-31

**Authors:** Baptiste Porte, Clémence Chatelain, Julie Hardouin, Céline Derambure, Yasmine Zerdoumi, Michèle Hauchecorne, Nicolas Dupré, Soumeya Bekri, Bruno Gonzalez, Stéphane Marret, Pascal Cosette, Philippe Leroux

**Affiliations:** 1 Normandie Université, UNIROUEN, U1245, INSERM, Institute for Research and Innovation in Biomedicine (IRIB), Rouen, France; 2 Normandie Université, UNIROUEN, UMR-6270, CNRS, IRIB, Mont-Saint-Aignan, France; 3 Normandie Université, UNIROUEN, Proteomic Facility PISSARO, IRIB, Mont-Saint-Aignan, France; 4 Normandie Université, UNIROUEN, UMR-S905, INSERM, IRIB, Rouen, France; 5 Metabolic Biochemistry Department, Rouen University Hospital, Rouen, France; 6 Neonatal Pediatrics and Intensive Care Department, Rouen University Hospital, Rouen, France; Hungarian Academy of Sciences, HUNGARY

## Abstract

Infants born before 29 weeks gestation incur a major risk of preterm encephalopathy and subependymal/intracerebral/intraventricular haemorrhage. In mice, an ontogenic window of haemorrhage risk was recorded up to 5 days after birth in serpine1 knock-out animals. Using proteome and transcriptome approaches in mouse forebrain microvessels, we previously described the remodelling of extracellular matrix and integrins likely strengthening the vascular wall between postnatal day 5 (P5) and P10. Haemorrhage is the ultimate outcome of vessel damage (i.e., during ischaemia), although discreet vessel insults may be involved in the aetiology of preterm encephalopathy. In this study, we examined proteins identified by mass spectrometry and segregating in gene ontology pathways in forebrain microvessels in P5, P10, and adult wild type mice. In parallel, comparative transcript levels were obtained using RNA hybridization microarrays and enriched biological pathways were extracted from genes exhibiting at least a two-fold change in expression. Five major biological functions were observed in those genes detected both as proteins and mRNA expression undergoing at least a two-fold change in expression in one or more age comparisons: energy metabolism, protein metabolism, antioxidant function, ion exchanges, and transport. Adult microvessels exhibited the highest protein and mRNA expression levels for a majority of genes. Energy metabolism–enriched gene ontology pathways pointed to the preferential occurrence of glycolysis in P5 microvessels cells versus P10 and adult preparations enriched in aerobic oxidative enzymes. Age-dependent levels of RNA coding transport proteins at the plasma membrane and mitochondria strengthened our findings based on protein data. The data suggest that immature microvessels have fewer energy supply alternatives to glycolysis than mature structures. In the context of high energy demand, this constraint might account for vascular damage and maintenance of the high bleeding occurrence in specific areas in immature brain.

## Introduction

Neurological defects are a major concern in neonatal care after preterm birth, *in utero* inflammation, birth asphyxia, or hypoxia-ischaemia episodes [1]. Patient age is a determinant factor of the topography of environmentally acquired injuries, because many brain development landmarks are completed during late gestational weeks and maturation is still ongoing at birth and in the postnatal period [2]. Vascular impairments have major neurological impacts in these fragile neonate populations, increasing the risks of cerebral palsy and behavioural and cognitive defects [3–6]. In extreme preterm infants (less than 28 completed weeks gestation), subependymal/intraventricular/ intraparenchymal brain haemorrhage is the most frequent cause of cerebral lesions, occurring in deep cerebral regions. The periventricular germinal matrix exhibits particular vulnerability, due to constitutive immaturity, high energy supply requirements due to angiogenesis, as well as neural precursor multiplication and hypoperfusion risk due to fluctuation of cerebral blood flow in sick infants [7]. Indeed, an immature cerebrovascular bed in periventricular germinative areas does not ensure autoregulation of cerebral perfusion, a self-protection capability acquired progressively during the third trimester of gestation [8–11]. The coincidence of weak autoregulation and fluctuant systemic blood pressure in extreme preterm infants represents a risk of pressure-passive perfusion, especially in the terminal vascular tree in periventricular areas. Arterial pressure variations could either produce transient high pressure perfusion or stasis periods, both of which have haemorrhage potential through mechanical or hypoxo-ischemic effects, respectively [12].

Neonate encephalopathy manifested as sensorial, motor, and/or cognitive defects affect patients who have experienced perinatal insults. An impaired development schedule in early infancy is one consequence, although brain lesions detected by medical imaging are not strictly correlated with lasting disabilities [1, 5]. Thus, besides acute vascular rupture resulting in bleeding, inconspicuous alterations of vascular function may affect the development of neural parenchyma, through alteration of the blood–brain barrier (BBB) in all its functions, including limitation of paracellular diffusion and changes in transendothelial transport and protein/xenobiotic exclusion. Furthermore, microvascular endothelium exert bidirectional signalling, the perturbation of which necessarily interferes in brain development and participates in pathological processes [13].

Although perivascular cell support is delayed in neonate periventricular areas, microvessels have non-permeant and insult-resistant BBB early in development [14, 15]. A characteristic of functional BBB is a high energy requirement in endothelial cells (ECs) to ensure transendothelial and exclusion transports [16]. Transcriptomic research directed on Slc family transporters at the BBB and the blood/cerebrospinal fluid barrier in choroid plexuses has been performed showing large expression increases between embryonic stages and adults, especially in choroid plexuses [17, 18]. However these studies did not focus on the postnatal period at high risk of haemorrhage. The present study was planned to explore the switch of this risk between P5 and P10 in mice.

During perinatal period, angiogenesis is another energy requirement for EC sprouting, navigation, and elongation [19]. Anaerobic glycolysis is the predominant ATP synthesis pathway in ECs, whereas mitochondrion oxidative phosphorylation and respiratory chain complexes play a lesser role in energy supply [20]. Moreover, the proliferative state in EC requires glycolysis enzyme PFKFB3 [21]. Angiogenic factors (VEGF, FGF2) impose high energy consumption in ECs and direct metabolism towards glycolysis, for instance by increasing Glut1 expression [21]. Conversely, lactate together with glucose provide an energy substrate in neonatal brain neurons [22–24]. Indeed, neonatal levels of circulating glucose increased with age, whereas lactate, hydroxybutyrate, and pyruvate regressed in most species [25]. Accordingly, the mono-carboxylate transporters (MCTs) and not the glucose transporter Glut1 predominate in rodent neonate brain vessels [26, 27], and MCT1 pharmacological blockade in neonatal mice has deleterious effects on neurons and glia [28]. The predominant expression of MCT1 and Glut1 was also observed *in vitro* in primary EC cultures [29]. It appears that ECs and neural cells have different needs with regard to energy substrate. The barrier position of ECs necessitates the expression of transporters to supply neural cells and domestic needs.

The strong dependence of ECs on glucose may become a weakness when steady-state conditions are challenged. Indeed, postischemic reperfusion and hypoglycaemia resulting in alternative oxidative pathways produce reactive oxygen species and immaturity of the antioxidant mechanism impairs redox potential [6, 19, 30]. Therefore, EC may be considered a possible Achilles’ heel in the immature brain when confronted with perinatal hazards given the frontline position of vessels towards environmental variations and their critical transport activities in determining nerve cells and progenitors’ fate.

We recently reported major remodelling of the vascular extracellular matrix (ECM) and extracellular matrix adhesion between postnatal day 5 (P5) and P10 in wild type C57Bl/6 mice, correlating with the haemorrhage propensity during this period in Serpine1 knock-out mice (PAI-1 coded by serpine1 gene is an inhibitor of plasminogen activator) [31, 32]. In the context of extreme preterm birth, bleeding occurs in immature germinative periventricular areas [1, 12]. The present work is a follow-up study designed to describe brain microvessel immaturity at the protein content and transcription regulation levels. The study was completed by forebrain microvessel (fMV) gene ontology on proteomic and transcriptomic data of enriched fMV from P5, P10, and adult mice, highlighting significant age-dependent variations in energy metabolism, transporters and antioxidant factors.

## Materials and methods

### Animals

C57Bl/6 and NMRI (Naval Medical Research Institute) mice were bred in the housing facilities of the Department of Medicine and Pharmacy of Normandy University and used according to the French law on ethics in experimental animal use (Articles R214-117 to R214-127 published on February 7, 2013) and the ARRIVE guidelines. Housing facilities authorization (B7645005) and protocol approvals (01680.02) were given by the French Ministry of Higher Education and Research on recommendation of local ethic committee (CENOMEXA; Comité d’Ethique Normandie en Matière d’Expérimentation Animale; n°54). Day of birth was considered day 1 of neonate life. Animals were kept at 21 ± 1°C with food and water *ad libitum* under a 07:00/19:00 h light/dark cycle. Pups were sacrificed for fMV extraction at P5 or P10 by decapitation, or at adulthood (P60 ± 5 days) by cervical dislocation under isoflurane anaesthesia. Since preterm bleeding occurs in periventricular area, we have dissected forebrains to collect periventricular germinative areas and choroid plexuses.

**Fig 1:**
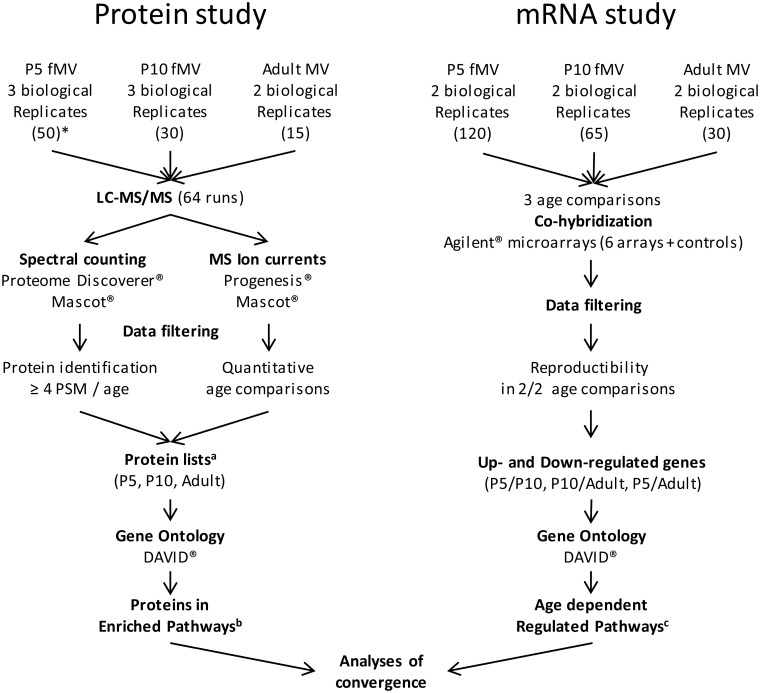
Experimental schedule of proteomic and transcriptomic approaches. * Numbers in parentheses indicate the number of pooled forebrains used for constitution of each biological replicate. ^a^ Protein lists are provided in S1 Table. ^b^ Proteins associated with KEGG pathways related to metabolism and transport are listed in S2 Table. ^c^ Age-dependent regulated pathways from the RNA hybridization assay are described in S3 Table.

Fifty, 30, and 15 forebrains (both sexes) of C57Bl/6 mice were used to construct P5, P10, and adult fMV protein samples, respectively. A total of 125, 65, and 30 forebrains were needed to collect a minimum of 224 ng fMV RNA from P5, P10, and adult NMRI mice. Three independent fMV isolations were done for protein studies at P5 and P10, and two preparations were made for adult proteins and for each RNA extraction. The experimental schedule is summarized in Fig 1.

### Forebrain microvessel extraction

fMV were obtained as previously described [31, 33]. Procedures were performed under RNase-free conditions. Dissected brains (*n* = 15 to 50 a day, depending on age) were sectioned posterior to the olfactory bulb and anterior to colliculi, and meninges were removed. Tissue homogenisation was performed in 7 mL complete MCDB (MCDB-131, GE Healthcare, Velizy, France) supplemented with 2% foetal calf serum, 100 UI/mL penicillin, 100μg/mL streptomycin, and 0.25 μg/mL fungizone using a Dounce homogenizer. The homogenate was rinsed in 50 mL buffer, resuspended in 50 mL complete MCDB containing 18% dextran, and centrifuged (30 min, 3200 rpm, 4°C). The supernatant was centrifuged again to improve recovery yield. The two pellets, suspended in 5 mL complete MCDB, were filtered through 70-μm mesh nylon membrane to remove debris. The filtrate was rinsed in 50 mL buffer and centrifuged (7 min, 1650 rpm, 4°C), and the pellet was suspended in 200 μL sterile phosphate buffered saline, dried, and frozen at –80°C until analysis. The purification of fMV was validated by the enrichment of vascular markers (Pecam and PDGF-Rβ) in fMV relative to raw brain homogenates [31]. Otherwise, the data strictly reproduce symetrical kinetics profiles of glucose and monocarboxylate transporters Glut-1 (Slc2a1 gene), MCT1 (Slc16a1 gene), increasing levels of the glutamate transporters EAAT2 and EAAT3 (coded by Slc1a2 and Slc1a1 genes, respectively) in adult compared to P10 vessels, while EAAT1 (Slc1a3 gene product) was constant [29, 34].

### Protein extraction

Protein extraction from microvessels was performed as described previously [31]. Dried fMV were suspended in 1 mL triethyl-ammonium bicarbonate buffer (0.2 M, pH 8.5) and solubilised in three steps: (i) a mild sonication (65 W, 20 kHz, 1 min; Vibra-Cell, Sonic Material Inc., Newtown, CT, USA) and centrifugation (15 min, 12,000 rpm, 4°C) provided the S0 fraction; (ii) a stronger sonication (104 W, 20 kHz, 1 min) of the pellet resuspended in 1 mL triethyl-ammonium bicarbonate buffer and centrifugation (15 min, 12,000 rpm, 4°C) provided the S1 fraction; (iii) the same strong sonication of the previous pellet resuspended in 1 mL of a highly denaturing buffer (urea 7 M, thiourea 2 M, tri-n-butylphosphine 0.05%, dithiothreitol 20 mM, C7BzO 0.5%, CHAPS 2%, SDS 1%) allowed complete dissolution, providing fraction S2. Due to low protein concentration in S0 from P5 samples, S0 and S1 were merged (v/v) into a so-called S01 combined fraction for each age. Small aliquots were used for protein determination by the Bradford technique.

### Tandem mass spectrometry

Proteins were treated and analysed by liquid chromatography and tandem mass spectrometry (LC-MS/MS) as previously described [31]. Briefly, 30 μg of protein per sample were reduced and alkylated (dithiothreitol and iodoacetamide) and concentrated by SDS-PAGE in a single thin band of protein revealed by Coomassie blue, lysed, and digested in gel by trypsin (0.1 μg/μL). Tryptic peptides were extracted with acidified acetonitrile water solutions, dried, and resuspended in formic acid (0.1%). Peptides (1 μg) were separated by nano-liquid chromatography (Easy-nLC II, Thermo Fisher Scientific, Villebon/Yvette, France), first on an enrichment column (Cap Trap C8, 0.5 × 2 mm, Michrom Bioresources, Auburn, CA, USA) followed by a reversed phase column (C18, L153, ID 5 μm, 100 Å pore size, Nikkyo Technos, Tokyo, Japan). The gradient (mobile phase A: H_2_O/0.1% formic acid; mobile phase B: CH_3_CN/0.1% formic acid) was delivered at 300 nL/min from 2 to 40% B in 105 min, then 40 to 80% B in 4 min, and a final step at 80% B for 15 min. The eluted peptides from C18 were injected into an LTQ-Orbitrap Elite (Thermo Fisher Scientific) mass spectrometer by electrospray ionisation at 1.5 kV and 200°C. Tandem MS/MS was performed in a data-dependent mode. The 20 most intense ions in full scan MS were selected (top 20 strategy) and fragmented by collision induced dissociation. MS and MS/MS analyses were done from 300 to 2000 m/z, and scan resolution was set at 60,000.

### Transcriptome analyses

Pelleted fMV suspended in lysis buffer with 1% β-mercaptoethanol were submitted to strong agitation with ceramic beads using a tissue lyser (Qiagen, Courtaboeuf, France) for cell disruption. RNA extraction was performed using the RNeasy Micro Kit (Qiagen) and labelled with either Cy3 or Cy5 fluorochromes then two by two cohybridised on whole mouse genome oligo 4x44K microarrays according to manufacturer’s indications and detailed in S1 File. Validation of the quantitative fluorochrome ratio for inter-age comparisons was performed by reverse fluorochrome labelling controls (dye swap assay), and validation of intergene expression levels was done on 14 genes showing low or high mRNA expression by multiplex PCR and hybridization data showing high correlation (*r*^2^ = 0.898) [31]. Data were in agreement with the Minimum Information on Microarray Experiment guidelines, deposited in the NCBI Gene Expression Omnibus (accession number GSE67870).

### Bioinformatics, biostatistics, and data management

Raw mass spectrometry files from LTQ-Orbitrap were analysed using Raw Meat software (version 2.1; Vast Scientific) to assess run quality. All raw files were analysed to describe protein profiles using Proteome Discoverer (v 1.3.0.339, Thermo Fisher Scientific) based on spectral counting (setting peptide confidence high). Inter-age quantitative comparisons were performed on fMV using Progenesis LC-MS (v 4.0.4441.29989, Nonlinear Dynamics). Quantification was based on extracted ionic currents with inter-age ANOVA statistical parameters set at *p* < 0.05, *q* < 0.05, and power > 0.8 at peptide and protein levels. The two software packages used Mascot (v 2.2, Matrix Sciences) with a cut-off identity score set at 25 for fMV, tryptic peptides, one missed cleavage allowed, and taking into account cysteine carbamidomethylation and methionine oxidation as variable modifications, according to the IPI database (version 20070119), fragment mass tolerance set at 10 ppm, and peptide tolerance set at 0.5 Da. Protein content was analysed from three independent fMV pools for P5 and P10 and two pools for adults. The criterion for protein recording, at a definite stage of development, was its occurrence after signal quality application in at least two of the three independent biological samples of fMV, using Proteome Discoverer. Subsequent relative quantitation of protein per sample, age, and fraction was performed using Progenesis. For any two-age comparison, all raw MS/MS files obtained were uploaded into the software. Reconstituted three-dimensional LC-MS/MS maps were aligned semi-automatically. A minimum of 84.1% and 70.9% overlap were observed for S01 and S2 analyses, respectively. Normalisation of isotopic signatures was achieved automatically on the entire map. A first ANOVA comparison was performed on peptide with the following cut-off parameters: *p* and *q* values < 0.05 and power > 0.8. Statistically filtered data allowed protein identification by Mascot (see above for identification parameters). Mascot decoy-basis searches provided false positive levels at a maximum of 5.48%. A second ANOVA analysis was done at protein levels with the same cut-off requirements. Conflicting peptides, showing sequences present in several related proteins, were excluded. Protein quantification was based on normalised cumulative abundances of the remaining peptides associated with a given protein. As protein extractions were performed on pooled enriched microvessels, normalization of abundances was done on total protein amount in each run. Data collected from Proteome Discoverer and Progenesis LC-MS allowed the compilation of protein lists at each stage.

In the transcriptomic study, hybridization microarrays were analysed using GeneSpring software (Agilent Technologies, Santa Clara, CA, USA). In each array, outlier spots and those with a heterogeneous signal on one colour were discarded. Spots exhibiting a Cy3/Cy5 fold change (|FC|) > 2 in the two experiments and *p* ≤ 0.05 were selected for analysis, excluding spots with hybridisation level < 1st percentile in the colour, or inter-array |FC| variation in each raw monochromatic signal > 50%. RNA samples from two independent experiments were hybridised on two distinct arrays for three comparisons, P5-Cy5/P10-Cy3, P10-Cy5/Ad-Cy3, P5-Cy5/Ad-Cy3. The three comparisons allowed discrimination of (i) early regulations (both up and down variations) observed in the P5-P10 comparison, noted as “early”; (ii) late regulations observed in the P10-adult comparison, noted as “late”; and (iii) long-term evolutions, observed in the P5-adult comparison. Slow evolutions referred to genes exhibiting P5-adult differences only. Because mRNA samples were obtained from dozens to hundreds of pups, one may consider that the requirement of inter-assay reproducibility allowed statistically valuable conclusions. Intensity ratios were analysed depending on variation in sense at the different ages. A total of 6018 probes fulfilled these criteria in at least one age comparison coding 5873 genes.

Protein content analyses were completed by KEGG pathway identification using the gene ontology online tool DAVID (DAVID Bioinformatics Resources 6.7, National Institute of Allergy and Infectious Diseases, National Institutes of Health) [35–37]. A Fisher’s exact test was used to assess KEGG pathway enrichment (threshold: *p* < 0.05). Protein lists were compared among samples, ages, fractions, and biological substrates using XL Comparator (online freeware, http://ccm.net/download/download-18158-xl-comparator), Excel-2010 (Microsoft, Issy les Moulineaux, France) and Venn diagram online (online freeware, Microarray Centre, CRP-Santé, Luxembourg). DAVID software was also applied to pooled up- and downregulated genes in each age comparison, using the data of the two replicates.

## Results

Protein detection by the top 20 MS/MS strategy allowed identification of 899 proteins, including 476 proteins (53% of total) observed at all ages and 29 (3%), 113 (13%), and 73 (8%) proteins observed exclusively in P5, P10, and adult, respectively, as previously described [31]. Forty-eight percent of the proteins were common to Peptide Spectrum Match (PSM) identification by Proteome Discoverer and comparative abundance analysis by Progenesis LC-MS. The former allowed comprehensive identification in separate samples, whereas the latter allowed precise quantification of ion current intensities in the same LC-MS/MS spectra–selected proteins, although only those exhibiting statistically relevant two-fold variations. Therefore, missing values in the result tables are not ambiguous because both were derived from the same MS runs although viewed using different filters. Protein or gene lists used for pathway analyses were based on protein identification regardless of software extraction and quantification. The use of comprehensive protein profiles for each age is, to our knowledge, a new approach in proteomic studies. Relatively few convergences between proteins and RNA variations were recorded, except those regarding cell adhesion and extracellular matrix reported earlier [31].

The 45 genes identified as having at least two-fold changes in protein levels and fluorescence hybridization signal in at least one age comparison can be classified into five major functions: energy metabolism, protein metabolism, antioxidant function, ion exchanges, and transport. Ionic currents, PSM and fluorescent signals are reported in Table 1.

**Table 1:** Convergence between regulated genes and proteins identified and quantified in P5, P10, and adult mice forebrain microvessels, according to pathways of interest.

UniProt	Gene ID	Gene regulation^a^			Protein quantification					
		Mean fluorescent signal (×10^3^)			Ionic current^b^ (×10^3^) Mean normalized abundance (Progenesis LC-MS analysis)			PSM^c^ (Proteome Discoverer analysis)		
		P5	P10	Adult	P5	P10	Adult	P5	P10	Adult
**Energy metabolism**										
P15327	Bpgm	**42.25±10.22** ^d^	6.55±0.92	3.66±0.12	-	-	-	**9**	8	
Q9WUA3	Pfkp	**5.55±0.63**	1.97±0.274	1.46±0.12	-	**1.82**	0.01	-	-	-
Q80XN0	Bdh1	**3.92±0.45**	3.53±0.51	1.81±0.29	3.02	**17.20±3.10**	3.89	-	-	-
P54869	Hmgcs2	1.74±0.12	**3.52±0.51**	1.81±0.29	0.10	**5.23**	-	-	**8**	-
O35459	Ech1	2.54±0.43	5.43±1.07	**5.52±0.34**	4.16±1.66^e^	**18.60±3**	9.50±3.70	-	**33**	-
P47738	Aldh2	2.46±0.13	4.49±0.41	**10.25±0.54**	2.21±2.17	3.99±1.75	**18.26±4.46**	-	37	**49**
P05064	Aldoa	52.08±16.82	52.08±5.97	**104.65±12.28**	92.35	113.05	**906.50**	33	53	**153**
**Protein metabolism**										
P62242	Rps8	0.12±0.02	**0.25±0.01**	0.23±0.01	-	-	-	-	16	-
P17156	Hspa2	1.03±0.07	1.55±0.09	**2.91±0.15**	-	-	-	**5**	-	-
Q64133	Maoa	1.07±0.094	2.43±0.32	**3.48±0.05**	2.52±1.19	**36.35±18.15**	10.13±7.67	-	**38**	-
Q8BW75	Maob	0.23±0.07	0.73±0.20	**3.53±0.13**	2.02	-	**18.10**	-	-	-
P15105	Glul	12.65±0.60	20.65±1.12	**54.53±0.93**	35.10	16.90	**42.25±18.65**	-	-	**14**
P63017	Hspa8	82.39±7.01	135.22±24.46	**194.88±17.16**	**1245.50±55.50**	345.50±86.50	247.25±183.15	149	**178**	138
A5JUZ1	Ubc	45.54±7.01	64.76±5.19	**123.23±12.62**	-	-	-	33	33	**37**
**Antioxidant mechanisms**										
Q61171	Prdx2	**85.23±11.90**	30.47±2.54	18.54±2.26	**358.00**	89.90	88.60	**55**	44	49
P11352	Gpx1	**81.79±9.71**	27.66±1.08	24.12±0.58	**43.40±14.50**	1.97	1.81	**12**	-	-
P24270	Cat	**10.02±2.25**	3.23±0.36	2.47±0.62	**82.50±20.50**	10.30	6.25	**21**	16	-
Q9DCN2	Cyb5r3	3.18±0.16	**6.75±0.87**	6.56±0.48	5.57±3.25	**33.60±20.30**	4.96±1.83	-	**46**	20
P19157	Gstp1	4.84±0.81	6.92±0.66	**9.91±1.01**	-	1.23	**6.95**	8	8	**27**
Q64444	Car4	8.90±0.86	10.28±0.62	**18.43±1.08**	1.37	47.00	**73.10**	-	15	**21**
P10649	Gstm1	6.18±0.84	7.66±0.61	**18.49±2.25**	4.30	5.07	**31.40±2.10**	-	-	**27**
**Ionic exchange**										
P13634	Car1	**0.37±0.02**	0.15±0.01	0.15±0.02	-	-	-	7	10	**22**
Q9WTT4	Atp6v1g2	0.34±0.02	0.37±0.02	**0.81±0.05**	-	-	-	-	-	**17**
P50516	Atp6v1a	3.24±0.12	4.30±0.51	**7.52±0.56**	**56.35**	8.58±2.83	2.61	18	15	**49**
Q6PIE5	Atp1a2	6.39±0.72	9.04±0.99	**14.13±0.69**	0.03	**13.43**	1.37	-	-	-
Q9JHL1	Slc9a3r2	5.22±0.82	10.59±1.22	24.53±1.31	4.43	-	22.90	-	4	-
**Transport**										
O35874	Slc1a4	7.55±0.55	4.17±1.14	1.48±0.28	1.09	17.60	-	-	-	-
P31648	Slc6a1	**6.63±0.42**	3.21±0.21	2.93±0.33	**6.75**	-	-	-	**4**	-
Q9Z2Z6	Slc25a20	0.26±0.06	**0.66±0.08**	0.41±0.03	-	**6.04**	1.85	-	-	-
P51881	Slc25a5	0.07±0.01	0.16±0.03	**0.27±0.02**	6.71±6.09	**49.20±1.40**	21.84±15.96	-	**11**	-
P49817	Cav1	0.43±0.09	1.15±.11	**1.41±.06**	7.35	45.80	**65.20**	-	-	**14**
Q9WVC3	Cav2	0.37±0.29	1.44±1.19	**5.59±0.20**	-	-	-	-	-	**6**
P43006	Slc1a2	4.31±0.25	5.98±1.17	**10.80±0.76**	6.88	11.20	**134.25±32.25**	-	4	**39**
P17809	Slc2a1	8.48±8.35	24.07±23.60	**122.92±4.27**	51.50±12.60	180.00±13	**869.00±16**	-	23	**50**
**Miscellaneous**										
Q62188	Dpysl3	**49.04±3.78**	18.20±0.59	2.99±0.049	**851.15±198.85**	158.7±49.7	61.10±49.90	**132**	109	67
P26645	Marcks	**23.56±1.69**	21.53±2.03	9.41±.027	**525.50±190.50**	51.40	31.80	**43**	17	-
Q6P1J1	Crmp1	**17.16±0.53**	7.74±0.75	1.99±0.053	**120.60±36.40**	16.24±6.86	13.28±12.02	**80**	76	37
Q91XF0	Pnpo	**14.19±3.46**	3.32±0.38	2.65±0.09	**32.30**	-	1.15	-	-	-
Q9EQF6	Dpysl5	**11.82±1.01**	4.26±1.09	2.58±0.39	**31.10**	-	3.61	**16**	4	-
O35098	Dpysl4	**11.11±0.91**	3.78±0.69	1.61±0.15	**30.70±9.10**	1.24	1.81	**22**	-	-
P30412	Ppic	14.52±1.38	**36.97±1.40**	6.28±0.43	1.33	**20.95±1.15**	0.68	-	**11**	-
A2AQR0	Gpd2	1.47±0.03	2.87±0.27	**4.73±0.10**	1.77±0.59	**55.45±5.45**	9.50±1.30	-	**28**	11
P21981	Tgm2	3.72±0.79	7.88±1.38	**11.03±0.23**	0.28	**24.90±8.80**	2.14	-	-	-
Q99L43	Cds2	0.61±0.05	0.87±0.11	**1.28±0.03**	0.89	-	**13.10**	-	4	**6**
Q8BVI4	Qdpr	3.86±0.28	5.80±0.42	**14.58±0.12**	-	-	-	-	-	**9**

PSM: peptide spectrum match.

^a^ Mean Cy3/Cy5 values based on genes with fold change (|FC|) > 2.

^b^ Selection was obtained using filters set as ANOVA *p* < 0.05, *q* < 0.05, power > 0.8 at peptide and protein levels.

^c^ Selection was obtained using filter sets on high peptide confidence.

^d^ SEM indicates variation of fluorescent signal.

^e^ SEM indicates variation of normalized abundance when protein was observed in two age comparisons.

### Gene ontology on forebrain microvessel proteome data

Ontology study of fMV proteins using the DAVID engine revealed 36 enriched pathways, with *p*-value < 0.05, in at least one developmental stage. Eight energy metabolism pathways (oxidative phosphorylation, citrate cycle, glycolysis/gluconeogenesis, pyruvate metabolism, pentose phosphate pathway, butanoate or propanoate metabolism, synthesis and degradation of ketone bodies, and fatty acid metabolism) and four amino acid metabolism pathways (arginine and proline, phenylalanine, tryptophan, and the aliphatic amino acids valine, leucine and isoleucine metabolisms) were identified, most of them at the three stages and highly significant (*p* < 0.001; Table 2). We focussed on proteins in these paths to determine whether they are constant constitutive elements or specific age-dependent factors (Fig A in S1 File, S1B Table).

**Table 2:** KEGG pathways significantly enriched in proteomes of P5, P10, and adult forebrain microvessels.

Forebrain microvessels		P5		P10		Adult	
KEGG term	Proteins in pathway	(n)	*p*-values*	(n)	*p*-values	(n)	*p*-values
Parkinson’s disease	133	39	3.40E-17	36	2.30E-13	**44**	**1.60E-20**
Huntington’s disease	183	43	4.40E-15	40	1.30E-11	**50**	**2.30E-19**
Oxidative phosphorylation	130	36	5.70E-15	34	3.40E-12	**42**	**3.60E-19**
Ribosome	89	28	3.60E-13	**36**	**1.30E-19**	24	1.50E-08
Alzheimer’s disease	182	37	4.90E-11	39	4.60E-11	**44**	**7.50E-15**
Citrate cycle (TCA cycle)	31	15	4.70E-10	**17**	**8.40E-12**	14	1.40E-08
Glycolysis / Gluconeogenesis	68	**18**	**3.80E-08**	18	8.90E-07	18	1.00E-07
Pyruvate metabolism	41	**12**	**1.60E-05**	11	2.10E-04	12	2.90E-05
Spliceosome	124	21	2.90E-05	**28**	**1.30E-08**	22	2.40E-05
Cardiac muscle contraction	78	16	3.70E-05	18	6.80E-06	**21**	**3.60E-08**
Butanoate metabolism	37	**10**	**2.10E-04**	8	8.50E-03	8	7.20E-03
Valine, leucine, and isoleucine degradation	46	**11**	**2.60E-04**	9	8.40E-03	8	2.30E-02
Amyotrophic lateral sclerosis	57	**10**	**5.50E-03**	10	1.00E-02	10	8.40E-03
Propanoate metabolism	30	**7**	**7.00E-03**	6	4.10E-02	6	3.70E-02
Phenylalanine metabolism	22	**6**	**7.80E-03**	5	5.00E-02	6	1.00E-02
Gap junction	86	12	1.10E-02	**19**	**6.70E-06**	15	8.20E-04
Prion diseases	35	7	1.50E-02	7	2.30E-02	**9**	**1.20E-03**
Tryptophan metabolism	40	**7**	**2.80E-02**	5	NS	6	NS
Nitrogen metabolism	23	**5**	**4.30E-02**	5	NS	5	NS
Synthesis and degradation of ketone bodies	10	**5**	**1.80E-03**	4	2.30E-02	3	NS
Fatty acid metabolism	45	**9**	**4.10E-03**	8	2.40E-02	6	NS
Systemic lupus erythematosus	103	11	NS	**15**	**6.10E-03**	**11**	NS
Long-term potentiation	72	6	NS	**11**	**1.40E-02**	**7**	NS
Aldosterone-regulated sodium reabsorption	42	3	NS	**8**	**1.70E-02**	**4**	NS
Melanogenesis	100	6	NS	**13**	**2.70E-02**	**8**	NS
Neurotrophin signalling pathway	130	11	NS	**15**	**4.00E-02**	**12**	NS
Proteasome	47	6	NS	**13**	**3.00E-05**	11	5.30E-04
SNARE interactions in vesicular transport	38	6	NS	**10**	**5.40E-04**	7	9.90E-03
Oocyte meiosis	115	14	NS	**17**	**2.80E-03**	15	1.20E-02
Tight junction	135	5	NS	16	**2.70E-02**	**17**	2.90E-02
Extracellular matrix-receptor interaction	83	9	NS	11	4.00E-02	**11**	**3.40E-02**
Vitamin B6 metabolism	23	1	NS	0	NS	**4**	**4.20E-03**
Fc gamma R-mediated phagocytosis	98	8	NS	10	NS	**14**	**7.80E-03**
Focal adhesion	198	15	NS	19	NS	**21**	**2.30E-02**
Arginine and proline metabolism	53	9	1.10E-02	8	NS	**10**	**4.60E-03**
Pentose phosphate pathway	26	5	1.60E-02	5	NS	**6**	**5.20E-03**

* *p*-values according to Fisher’s exact test. Boldface indicates the highest protein numbers and the lowest *p*-values between ages. (n) indicates the number of protein detected in the pathway.

Several pathways were related to protein synthesis and degradation activity (spliceosome, ribosome, and proteasome), and antioxidant pathways also exhibited significant enrichment. The comprehensive lists of pathways and associated proteins are provided in S1A Table. Some of them were not significantly enriched at all stages, although some associated proteins were in fact detected. Details are provided in S1B Table.

#### Energy metabolism associated proteins

**Fig 2:**
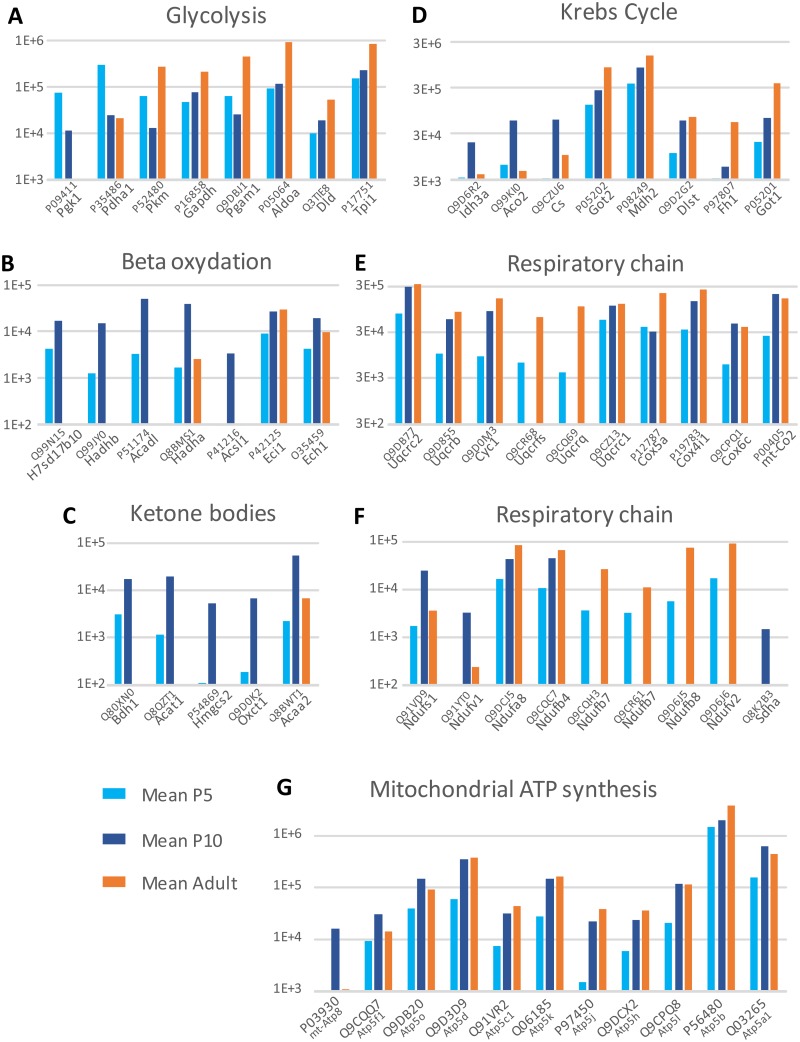
Development profile of energy metabolism proteins identified by LC-MS/MS in forebrain microvessels at three ages and involved in glycolysis (A), lipid beta-oxidation (B), ketone bodies metabolism (C), Krebs cycle (D), and respiratory chain (E, F). Mean Progenesis ionic currents are plotted at a log scale.

Eighty-eight proteins associated with energy metabolism, defined by DAVID pathways, were observed by LC-MS/MS analysis of fMV (see detection and comparative analysis details in S2A Table). Of them, 46 proteins exhibited the highest abundance in adult fMV, whereas 35 proteins were predominant in P10 fMV and only 7 proteins in P5 fMV. The proteins that exhibited their maximum expression at P5 are involved in glycolysis (e.g., phosphoglycerate kinase, pyruvate dehydrogenases, pyruvate kinase, glyceraldehyde phosphate dehydrogenase). Maximum abundance at P10 was observed for proteins involved in the metabolism of ketone bodies, beta oxidation, and to a lesser extent the Krebs cycle. Respiratory chain–associated proteins had maximum expression in adult (27/39) or at P10 (12/39). The majority of mitochondrial ATP synthases (8/11) increased after P5 to reach their adult level as early as P10 (Fig 2). Although the top 20 strategy of detection allowed partial description of these pathways, usually 20–54% of recorded genes, data indicate a shift of energy metabolism effectors from glycolysis to oxidative phosphorylation pathways between P5 and P10, which was further reinforced in adult fMV.

#### Transport proteins

**Fig 3:**
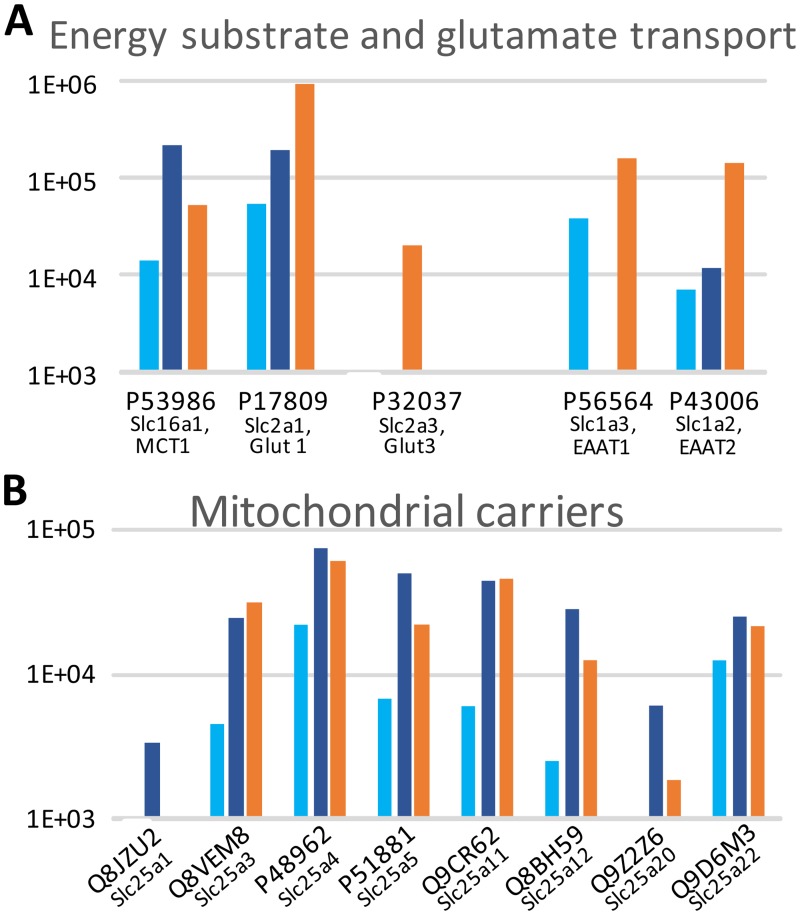
Developmental pattern of transport proteins in P5, P10, and adult (Ad) mice brain microvessels. **A**; Energy substrate and glutamate transport, **B**; Mitochondrial transporters. Bar colour code as in Fig 2.

No gene ontology pathway describes endothelial transport in the mouse. Since transendothelial transport is an important function of the BBB, we isolated solute ligand carriers (Slc) and ATP-binding cassette transporters (Abc) in protein data to examine the changes that eventually occur during development (Fig 3, S2B Table, Fig A in S1 File). Thirty-eight proteins (Slc, Abc) segregating in 18 ion exchangers and 20 carriers of amino acids, energy substrate or neurotransmitters, and two caveolins were identified in fMV extracts. Energy metabolism transporters appeared in this top 20 LC-MS/MS identification strategy. The glucose transporter Glut1 (Slc2a1) exhibited a progressive rise from P5 to P10 (× 3.5) and then from P10 to adult (× 4.8). Glut3 (Slc2a3) had the same kinetics, although at a lower level. The monocarboxylate transporter MCT1 exhibited a transient high level at P10 followed by a large decrease in adult fMV (Fig 3A).

Several mitochondrial transporters (Slc25 family) were detected with a transient high at P10 (ATP/ADP exchanger Slc25a4, Slc25a5; carnitine/acylcarnitine carrier Slc25a20) or late increase (glutamate carrier Slc25a22). Of note, the mitochondrial shuttles for aspartate/malate (Slc25a12) and to a larger extent oxoglutarate/ketoglutarate (Slc25a11) allowing mitochondrial respiratory chain function were low at P5 and underwent large increase reaching their adult level at P10 (Fig 3B). Similar increasing kinetics was observed for the mitochondrial phosphate carrier (Slc25a3). These data suggest a progressive predominance of mitochondrial energy metabolism from P10 onwards.

Glutamate transporters associated with glutamate uptake and involved in synaptic clearance (EAAT1 and EAAT2) increased with development (Fig 3A). Conversely, low levels of GABA transporters (GAT1) were detected at P10 preferentially. Transcytosis-associated proteins, caveolin 1 and 2, and the secretory carriers (Scamp1 and Sc3) exhibited an increase to their highest level in adult fMV (S2B Table).

#### Antioxidant metabolism associated proteins

Twenty-two proteins involved in antioxidant activity were detected in fMV. Notably few proteins were predominant in P5 fMV (peroxyredoxin-2, peroxyredoxin-6, glutathione peroxidase-1, catalase, and glutathione S-transferase omega-1; S2C Table). Reciprocally, peroxyredoxin-1 and -5, several glutathione S-transferases, and superoxide dismutases reached their maximum in adults. Peroxyredoxin-6 exhibited a transient low level at P10. These few proteins associated with glutathione metabolism indicate the substitution of several antioxidant factors during development. It is difficult to state whether the diversification of actors increases antioxidant potential with maturity in fMV.

#### Protein biosynthesis–and degradation–associated proteins

Ninety-seven proteins included in the KEGG pathways and related to protein biosynthesis and degradation (ribosome, spliceosome, and proteasome) were detected at least at one age in fMV (Table 2). The vast majority of genes associated with splicing, ribosomes, post-translation metabolism, and proteasome had transient maximum level in P10 fMV, likely due to intense biosynthetic activity at this stage (S2D Table).

### Transcriptome analysis in microvessels

An ontology study of the regulation of fMV transcripts (|FC| ≥ 2) using DAVID revealed 56 significantly enriched pathways (*p* < 0.05) in at least one two-age comparison. Contrary to protein-based ontogeny, transcription-based pathway analysis did not converge towards energy metabolism pathways or protein metabolism pathways. This mismatch could reflect the fact that transcription analysis was based on time-dependent changes regardless of expression levels. Enriched pathways were distributed in seven groups (in decreasing order of significance): cell adhesion, development and morphogenesis, cancer, signalling, vascular functions and pathology, immunity, and metabolism (S3A Table). The major regulations related to extracellular components and cell adhesion have been discussed previously [31].

**Fig 4:**
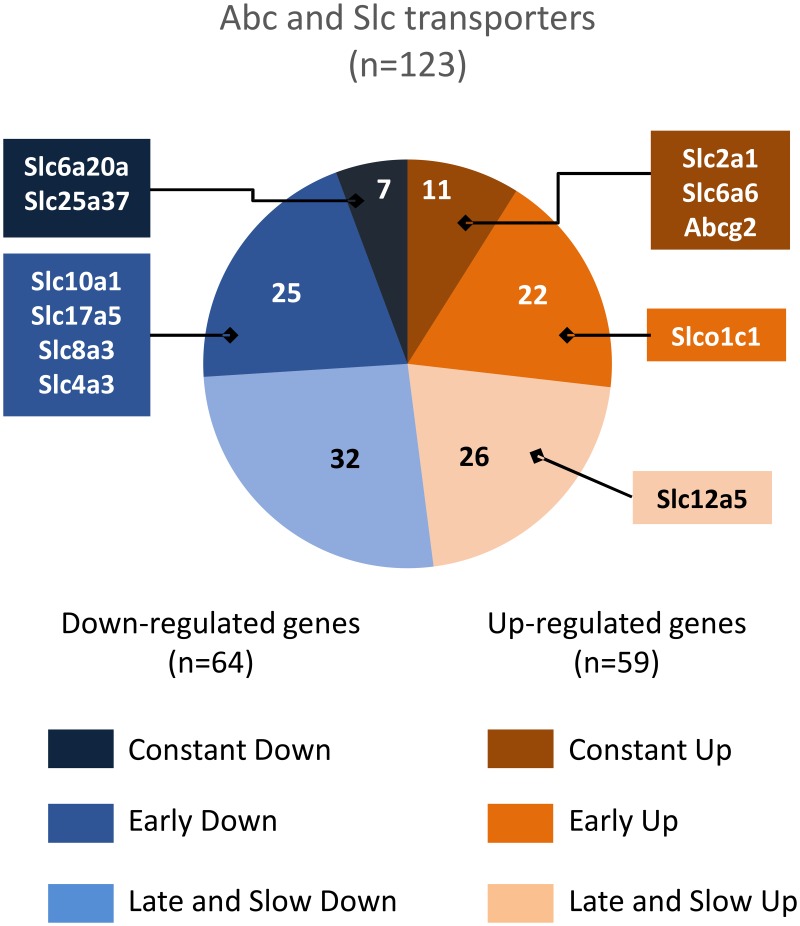
Ontogenic expression pattern of 138 significantly regulated genes coding for Abc and Slc transporters. Colour-coded sectors refer to developmental pattern. *n* in sectors indicates the total number of genes in the group. Boxes mark genes in each group in the top 10 highest expression level of all transcripts in the arrays. Slc2a1, solute carrier family 2 (facilitated glucose transporter) member 1 (Glut1); Slc6a6, solute carrier family 6 (neurotransmitter transporter taurine) member 6 (Taut); Abcg2, ATP-binding cassette, subfamily G member 2 (MXR1); Slco1c1, solute carrier organic anion transporter family, member 1c1 (OATP2); Slc12a5, solute carrier family 12, member 5 (KCC2); Slc6a20a, solute carrier family 6 (amino acid transporter) member 20A (Xt3s1); Slc25a37, solute carrier family 25 member 37 (mitoferrin); Slc10a1, solute carrier family 10 (sodium/bile acid cotransporter family), member 1 (Ntcp); Slc17a5, solute carrier family 17 (anion/sugar transporter) member 5 (Sialin); Slc8a3, solute carrier family 8 (sodium/calcium exchanger) member 3 (Ncx3); Slc4a3, solute carrier family 4 (anion exchanger) member 3 (Ae3).

Enriched pathways related to development, morphogenesis, cancer, signalling, and immunity mainly emerged from the P5-adult comparison, indicating that these functions evolve slowly during the entire P5 to adulthood period. Conversely, pathways related to vascular function including motricity or vascular pathology were most represented in the P5-P10 comparative analysis, or in the long-term interval, but far less in the P10-adult period (e.g., vascular smooth muscle contraction, dilated cardiomyopathy, or arhythmogenic right ventricular cardiomyopathy), indicating that major development of vascular functions occurs in the P5-P10 period. Leucocyte transendothelial migration, Abc transport, and endocytosis also included many genes with P5-P10 regulation (S3A and S3B Table). Indeed, 128 Slc and Abc transporter transcripts exhibited at least a two-fold change in expression (62 up- and 66 downregulations) in at least one time interval (S3B Table). Similar numbers of genes were up- or downregulated in the P5-P10 period, although upregulations mainly involved highly expressed genes (Fig 4). The energy supply transporters Glut1 and Mct5 exhibited high expression levels and constant increases over time. Reciprocally, Mct10, Mct2, Mct14, and Glut9 exhibiting lower expression levels underwent time-dependent decreases. The fatty acid transporters Slc27a1, Slc27a4, and Slc27a5 showed early and lasting downregulations. These observations pointed to energy substrate transport alternatives to glucose at earlier stages. Surprisingly, no change in expression level was noticed for the aspartate/malate mitochondrial shuttles genes (Slc25a11 and Slc25a12).

Many BBB efflux protein transcripts exhibited high amplitude developmental upregulations (FC up to almost 10) at high transcription levels (Mxr1, P-gP, Mrp4, Mrp6, Tap1, Tap2, Mdr1, and Mdr2). Some downregulations were also observed (Cftr, Mrp5), although usually at lower expression levels (Abcc8, Abcg4, and Abcg5). Many transcripts for ions, amino acids, neurotransmitters, and metabolite transporters at the plasma membrane or mitochondria exhibited reciprocal regulations indicating substitution of many effectors during development (S3B Table). These observations suggest that, although the BBB is considered closed at early development stages, its exclusion function remains immature in postnatal mice.

Few metabolism-related pathways were isolated, mainly in one time interval and with low significance. DNA metabolism–related genes exhibited downregulation in line with the cessation of growth in adults. Conversely, sphingolipid metabolism–related genes mostly exhibited a constant rise in expression levels. Genes associated in the drug metabolism and the glutathione metabolism KEGG pathways appeared mainly at late onset, with many more genes associated with glutathione metabolism upregulated during development. However, the few downregulated genes had the highest expression levels. The transcripts of Gpx1, Gstm1, and Gstp1 whose proteins were identified reproduced the protein kinetics (S3C Table). Our data suggest that antioxidant function is assumed by a higher diversity of actors in mature than in immature tissues, likely indicating a lower cytoprotection potential in neonatal than in adult microvessels.

No energy metabolism pathway was extracted based on age variation of transcript levels (S3C Table).

## Discussion

The present work extends and completes our previous study demonstrating the remodelling of fMV between P5 and P10 [31]. The findings regarding extracellular matrix, adhesion molecules, and integrins strongly suggested the improvement of vascular strength that may reduce haemorrhage risk in this period. Validation of proteomics and transcriptomics methodology, data management, and statistical approaches were discussed in detail [31]. The present data based on an ontology approach identifies the shift of energy metabolism, transport, and antioxidant effector proteins during development indicative of age-dependent maturity features.

Gene ontology extraction of enriched pathways allowed us to compare three development stages based on proteins and transcript data with distinct sensitivities and constraints. Protein detection was based on the highest abundance at one stage, whereas gene transcription was evaluated as dynamic interstage comparisons, regardless of expression level. Although our proteomic study detected many pathways related to energy metabolism and protein biosynthesis and degradation, transcript analysis did not converge towards these functions. This apparent discrepancy likely reflects differences in statistical methodology. In the proteomic study, the top 20 LC-MS/MS strategy revealed the most abundant genes and isolated enriched pathways within a subset of proteins actually identified, whereas transcript analyses referred to the full genome. The low level of convergence may also reflect biological differences in, for example, proteins and mRNA turnover. Nevertheless, transcriptomics evolution data strengthened the proteomic data at the individual gene level for a large number of genes related to energy metabolism (Hmgcs2, Aldoa, Aldh2), antioxidant activity (Prdx2, Gpx1, Cat), and transport (Slc2a1 (Glut1), Slc1a2 (Eaat1) or caveolins).

Several enriched pathways extracted from proteome data exhibited convergences with reported analyses of adult brain vessels proteome [13, 38, 39], BBB transcriptome pathways [17, 18], choroid plexus transcriptome [18, 40]. Indeed high convergence with the literature was noted for adhesion and basal lamina proteins [31, 38]. In addition, 34 transport proteins in the 36 identified were common to the near hundred transporters and channel observed in adult microvessel membranes (S2B Table) [38]. Their vast majority (32/36) was observed in adult fMV. The only transport protein absent in adult fMV were Atp6v1c1 (a lyzosomial proton transporter), and Slc1a4 (SATT, a glutamate and neutral aminoacid transporter), Slc25a1 (a mitochondrial citrate transporter associated to glycolysis/neoglucogenesis pathway) and Slc6a11 (GAT3, a neurotransmitter GABA transporter). None of the 34 identified proteins exhibited transcription down regulation while seven of them showed 2–8 fold P5-adult transcription up regulation in microarrays namely (Atp1a2 (NA/K ATPase), Slc1a2 (EAAT2 excitatory aminoacid transporter), Slc25a5 (mitochondrial adenine transporter), Atp6v1a (lysosomal H+ transporter), Slc2a1 (Glut1 glucose transporter), Slc9a3r2 (plasma membrane H+/Na+ exchanger), Slc25a20 (Carnitine/acylcarnitine translocase)).

Transcriptomic approach allowed more comparison on transport molecules with reports from the literature, although they were often obtained on different biological material and different ages. We detected 123 gene transcriptomic regulations including 105 Slc genes while 216 Slc-coding transporter genes were observed by Saunders and co-workers in choroid plexus and/or endothelial parenchymal cells [18]. Only few (17) entities showed parallel age dependent evolution between our data and this study which considered foetus and adult stages while we compared P5/P10 and adult microvessels. Of note only two showed divergent evolution; Slc6a6 (taurine transporter) and Slc39a10 (Zn transporter) [18]. Both cerebral endothelial cells or by choroid plexus epithelial cells were present in our fmV extracts although in unknown proportions. The majority (71%) of the 105 Slc regulated transcripts we showed were mentioned in endothelial cell and/or choroid plexus cell lists [18]. Thirty-four genes were expressed in both tissues and 19 genes appeared specific to endothelial cells. The expression of 22 genes in choroid plexus cells is indicative of a significant contribution of choroid plexuses in our extracts. Indeed, 7 genes in our study underwent parallel kinetics in a gene expression study of the choroid plexus from P2 to Adult, (Abcc4, Slc22a8, Slco1a5, Slco1c1, Slco2a1). In another study, convergent data were noted on the antioxidant protein coding genes (Sod3, Maob, Ddc and Cat) [40].

Using a different database (Ingenuity pathway analysis) convergent lists of enriched pathways were obtained, related to Glycolysis/neoglucogenesis, phenylalanine metabolism Wnt, IL1, or nuclear receptor signalling [17]. Other reports using different approaches to extract the core of endothelial secreted proteins from 4 independent studies identified a “vasculome” of 99 proteins from adult mouse brain microvessels [13]. Near 60% of these proteins were identified in forebrain enriched microvessels as proteins (n = 22) or exhibiting mRNA expression modulation (n = 42). In brain capillaries study using multiple reaction monitoring (MRM) and in silico selection of peptides prior to MS/MS identification, 12 proteins were detected in adult mice preparations [39]. Using a top20 MS/MS strategy, we detected 3 of these proteins (Slc2a1 (Glut-1), Slc16a1 (MCT-1) and Slc7a5 (Lat1)) and 5 more genes exhibited ontogenic transcription up regulation (Abcg2, Slc22a8 (Oat3), Slco1b2 (Oatp2), Slc6a6 (Taut) and Ggt1). Altogether, our data are in accordance with the literature. Studies of pure cell population in culture, extracted membrane fractions or using MRM approaches were more sensitive that the present combination of raw material and the top 20 proteome strategy that reciprocally illustrated the physiology of microvessels during ontogeny, taking account for their complex cell content and microenvironment.

Vascular weakness, erratic haemodynamics, and coagulation deficiency are common hypotheses invoked to explain the high rate of subependymal/intraventricular/intraparenchymal brain haemorrhage in early preterm infants [12]. Vessel rupture is the nadir of vascular damage but it is likely that silent endothelial dysfunctions affect neonate brain development even when objective imaging of lesions are lacking to explain preterm encephalopathies. The present data indicated a glucose-centred energetic dependence in microvessels at P5, principally based on the observation of alternative pathway components from P10 onwards; that is, the late onsets at P10 of the mitochondrial respiratory chain proteins or oxoglutarate/ketoglutarate and malate/aspartate shuttles of mitochondrial membrane indicate that a low proportion of the energy supply is based on the respiratory chain at P5. From P10 onwards also, lactate, oxidative phosphorylation, pentose phosphate, and ketone bodies degradation may support endothelium energy supplies. The view of early glycolysis dependence of developing vasculature is in concordance with the glycolysis-dependent BBB onset that essentially takes place during foetal life, is still ongoing with brain growth during the early postnatal period, and is completed before P10 in rodents [23]. This suggests that neonate microvessels have fewer alternatives than mature structures for energy supplies. Together with delayed extracellular matrix development and cell adhesion immaturity, such energetics’ inability to cope with hypoglycaemia likely contributes to vascular vulnerability early in development in a setting of environmental disturbances (e.g., hypoxia-ischaemia). In fact, the hypoxia alteration of VEGF-dependent angiogenesis in periventricular germinal matrix determines the ultimate pattern of periventricular leukomalacia in mice [6, 30]. Anti-VEGF therapeutic manipulation may have a deleterious outcome in human preterm retinopathy [41]. Indeed, energy metabolism in ECs changes throughout the angiogenic process [19]. During development, tube extension appears to be highly glucose dependent at the tip leader cells, whereas postmitotic ECs in neotubes reduce their demand only to maintain homeostatic functions [19]. In fact, glycolysis experimental inhibition promoted quiescence [21]. Our observation of enriched glycolysis factors relative to other ATP-generating pathways in P5 pooled fMV is in accord with this view from the literature of predominant glycolytic activity at the earliest developmental stages. The concept of a glycolysis-based energy metabolism in ECs is also supported by their low density of mitochondria and their marginal contribution to the energy supply [42]. Our data revealing late onset of respiratory chain components and of many mitochondrial transporters may also account for the relatively minor mitochondrial role in neonatal than in adult EC energy metabolism.

The predominance of monocarboxylate transporters compared to glucose transporters observed here and described in the literature at early development stages may appear to be discrepant with glucose EC dependence [26, 28, 29]. However, neuronal activity in early development has been shown to depend on lactate, or the ketone body hydroxybutyrate, in neonatal but not in adult hippocampus [22, 43, 44]. Because the astroglial population is sparse at P5 and likely accounts for a weak supply of lactate, circulating monocarboxylates may be the source of these energy supplies [25]. The localization of MCT1 in the vascular wall of rat neonate in the first postnatal week is in line with the blood supply of monocarboxylates to brain parenchyma [26]. We previously reported that P10 EC cultures had a high Mct1/Glut1 ratio, which was opposite of the adult EC pattern [29]. The presence in microvessels of the highest Mct1 protein level at P10 and the constant or late decreases of Mct2, Mct10, and Mct14 mRNAs more likely account for neuron monocarboxylate-based metabolism than for EC needs [25] and are indicative of the transport function of EC at the immature BBB. Alternatively, a contribution of Mct(s) to vessel energy metabolism by providing substrates for neoglucogenesis cannot be excluded [25]. Lactate promotes barrier genesis and mitochondrial replication activities in vessels, which are dependent on metabolic effects and receptor activation, respectively [23, 45].

Cortical development includes concomitant vascular tree development and BBB acquisition. Time windows of neonate brain vulnerability were shown to depend on vessel maturity [6]. In addition to evidence of strengthening the extracellular matrix and improving EC intercellular adhesion and adhesion to basal lamina between P5 and P10 reported earlier [31], the present data provide new information on BBB function. The sustained and high-amplitude rise observed in mRNA levels for the major exclusion proteins, P-gp (Abcb1a) and BCRP (Abcg2) is in accord with data in rats [46] and may account for the poor ability to exclude xenobiotics at the neonate mouse BBB. However, reciprocal kinetics was also observed and suggests age-specific exclusion properties of the brain vessels rather than immaturity of the function [47]. Protein identification at specific sites (i.e., choroid plexuses or meninges) is the next task in this research.

Glycolysis is considered a safe energy source because anaerobic function produces few reactive oxygen species. The risk of oxidative stress is especially acute in vessels where oxygen pressure is high and especially in neonate periventricular areas with fluctuant perfusion. Although cortical development of P5 mice is somewhat analogous to human foetus (or preterm) at 30 weeks gestation, neonatal mice are adapted to the extra-uterine environment. Antioxidant proteins were in fact present early and in high levels in neonate mice. Although the diversity rises with age, low anti-oxidant potential in extreme preterm may affect vessel integrity in the context of postnatal hyperoxic stress [48]. Therefore exploration of antioxidant strategies in human extreme preterm is necessary in the approach of vasculoprotection. Indeed, late gestation adaptations are likely to occur, as reported in umbilical cord ECs [49]. Whereas preterm cord ECs have high oxidative phosphorylation, generating reactive oxygen species, the metabolism in term neonate cord ECs has turned towards glycolysis, a protective strategy to cope with birth hypoxic conditions. The correlate is less oxidative stress in term than in preterm cord ECs. Whether this concept may be translated to brain requires further investigation in human since, to our knowledge, there is no reports in the literature describing antioxidant protein ontogeny.

Our aim in this study was to isolate putative transient factors of risk for bleeding in neonatal fMV in mice, at comparable development stages of risk of bleeding and encephalopathy in human preterm infants (aged less than 28 completed gestation weeks). Restricted alternatives for energy supplies in the early postnatal period observed in mice, concomitant with specific anti-oxidant and exclusion protein expression patterns, may be vulnerability factors towards genetic deficiencies or environment disturbances at this time. Human correlates are now required to corroborate these data obtained in mice and to develop nutritional actions, for instance, to prevent vascular damage in preterm neonate populations, keeping in mind that vessels and neural cells likely have distinct needs.

## Supporting information

S1 File Detailled material and methods on transcriptome analyses, transcriptome analyses methodology and validation. List and captions of supplement tables. Supporting fig A. Venn charts of pathway-associated proteins detected in P5, P10, and in adult (Ad) mice brain microvessels. (A) Distribution of proteins associated with energy metabolism: oxidative phosphorylation, citrate cycle (TCA cycle), glycolysis/gluconeogenesis, pyruvate metabolism, butanoate metabolism, propanoate metabolism, synthesis and degradation of ketone bodies, fatty acid metabolism, and pentose phosphate KEGG pathways. (B) Protein metabolism including ribosome, spliceosome, proteasome, phenylalanine metabolism, arginine and proline metabolism, tryptophan metabolism KEGG pathways. (C) Antioxidant mechanisms including peroxiredoxins, thioredoxins, and superoxide dismutase. (D) Transport including Slc, Abc, and Cav gene–coded proteins.
(DOCX)

S2 File Porte et al. journal of cerebral blood flow and metabolism. Copy of the first part of the study [31].
(PDF)

S3 File Animal use authorization. (PDF)

S1 Table Proteins identified in forebrain microvessels at the three ages. **S1A**; KEGG pathways statistically enriched in microvessel proteins identified by LC-MS/MS, according to DAVID freeware, **S1B**; Lists of proteins in all pathways at the three ages.
(XLSX)

S2 Table Microvessel protein lists in KEGG pathways related to metabolism and transport. **S2A**; Energy metabolism–associated proteins in forebrain microvessels, **S2B**; Transport associated–proteins in forebrain microvessels, **S2C**; Antioxidant metabolism–associated proteins in forebrain microvessels, **S2D**; Protein metabolism–associated proteins in forebrain microvessels.
(XLSX)

S3 Table Forebrain microvessels transcriptomic age differences highlights. **S3A**; DAVID-enriched pathways derived from comparative analyses of forebrain microvessels from mice pups at 5 days, 10 days, or adults, **S3B**; Detailed gene transcription levels of transport Slc and Abc genes, **S3C**; Detailed gene transcription levels in specific DAVID pathways associated with vascular function and metabolism.
(XLSX)
