# Supplementary material for: Proteomic and transcriptomic study of brain microvessels in neonatal and adult mice
Source: PLoS One. 2017 Jan 31;12(1):e0171048. doi: 10.1371/journal.pone.0171048 (PMC5283732; doi:10.1371/journal.pone.0171048)
Supplement: S1 File — List and captions of supplement tables. Supporting fig A. Venn charts of pathway-associated proteins detected in P5, P10, and in adult (Ad) mice brain microvessels. (A) Distribution of proteins associated with energy metabolism: oxidative phosphorylation, citrate cycle (TCA cycle), glycolysis/gluconeogenesis, pyruvate metabolism, butanoate metabolism, propanoate metabolism, synthesis and degradation of ketone bodies, fatty acid metabolism, and pentose phosphate KEGG pathways. (B) Protein metabolism including ribosome, spliceosome, proteasome, phenylalanine metabolism, arginine and proline metabolism, tryptophan metabolism KEGG pathways. (C) Antioxidant mechanisms including peroxiredoxins, thioredoxins, and superoxide dismutase. (D) Transport including Slc, Abc, and Cav gene–coded proteins. (DOCX) [file pone.0171048.s001.docx]

**Porte et al., S1 Supporting information**

**Transcriptome analyses**

Pelleted fMV were suspended in lysis buffer with 1% β-mercaptoethanol and submitted to two runs of strong agitation (16 s at 50 Hz) with ceramic beads (1.4 mm, Ozyme, Montigny-le-Bretonneux, France) using a tissue lyser (Qiagen, Courtaboeuf, France) for total cell disruption. Total RNA extraction was performed using the RNeasy Micro Kit (Qiagen), with a 260/280 integrity ratio above 1.9 and an RNA integrity number above 9.0, using a Nanodrop 2000c spectrometer (Thermo Fisher Scientific, Waltham, MA, USA).

Two-colour comparative hybridization was performed using a Whole Mouse Genome Oligo 4×44K Microarray (G4845A, Agilent Technologies, Les Ulis, France) to compare gene expression profiling in fMV from P5, P10, and adult mice. cRNA was synthesised from 100 ng total RNA and labelled using a Quick Amp Labelling Kit (Agilent Technologies). A total of 825 ng of cRNA was cohybridised on microarrays for 17 h at 65 °C. Raw hybridisation data evaluated on 5-μm pixel size using an Agilent DNA microarray scanner (G2565CA, Agilent Technologies) were extracted and normalised, then transferred to GeneSpring (GX 12.6 software, Agilent Technologies) for data processing and data mining. All profiling of the three age comparisons was performed in two biological replicates. A Dye Swap assay made of reciprocally Cy3/Cy5-labelled P10 and adult RNA was included in each experiment.

**Transcriptome analyses validations**

Dye Swap assays using reciprocal Cy labelling of P10 and adult material revealed linear correlations with a slope close to 1 and high correlation, ensuring that stage differences in expression levels did not result from differential cyanine labelling efficiencies. High correlations of P10-Cy3/Ad-Cy5 and P10-Cy5/Ad-Cy3 assays were observed considering fold change or normalised ln(FC) ratios. In addition, quantitative multiplex polymerase chain reaction performed on selected genes confirmed the FC measured in arrays in the three comparisons [1].

**List of supplementary tables** (provided off line in separate Excel files)

**S1 Table**: Proteins identified in forebrain microvessels at the three ages

**S1.1**: KEGG pathways statistically enriched in microvessel proteins identified by LC-MS/MS, according to DAVID freeware

**S1.2**: Lists of proteins in all pathways at the three ages

**S2 Table**: Microvessel protein lists in KEGG pathways related to metabolism and transport

**S2.1**: Energy metabolism–associated proteins in forebrain microvessels

**S2.2**: Transport associated–proteins in forebrain microvessels

**S2.3**: Antioxidant metabolism–associated proteins in forebrain microvessels

**S2.4**: Protein metabolism–associated proteins in forebrain microvessels

**S3 Table:** Transcriptomic study in forebrain microvessels at the three ages

**S3.1**: DAVID-enriched pathways derived from comparative analyses of forebrain microvessels from mice pups at 5 days, 10 days, or adults.

**S3.2**: Detailed gene transcription levels of transport Slc and Abc genes

**S3.3**: Detailed gene transcription levels in specific DAVID pathways associated with vascular function and metabolism

**Supplemental Reference**

1. Porte B, Hardouin J, Zerdoumi Y, Leroux P. Major remodeling of brain microvessels during neonatal period in the mouse: a proteomic and transcriptomic study. *J Cereb Blood Flow Metab* 2016; doi: 10.1177/0271678X16630557.

**A : Energy metabolism**

**B : Protein metabolism**


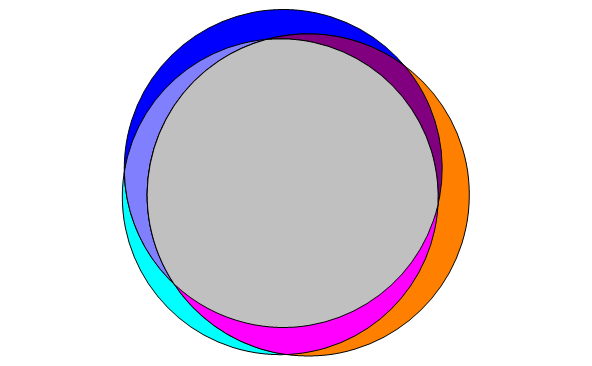


**61**

**5**

**4**

**7**

**1**

**5**

**5**

P5-specific proteins

P10-specific proteins

Ad-specific proteins

Proteins common to P5&P10

Proteins common to P10&Ad

Proteins common to P5&Ad

Proteins common to all stages


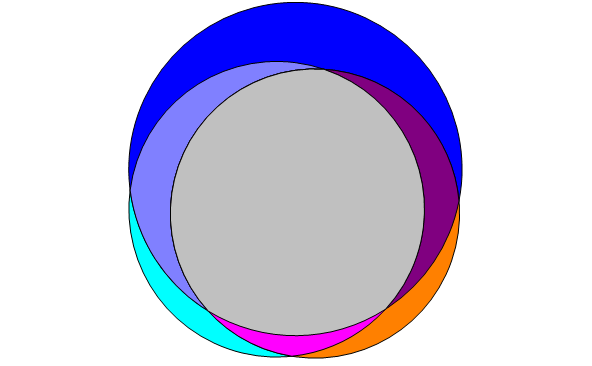


**53**

**11**

**8**

**4**

**2**

**2**

**17**

**C : Antioxidant metabolism**

**D : Transport**


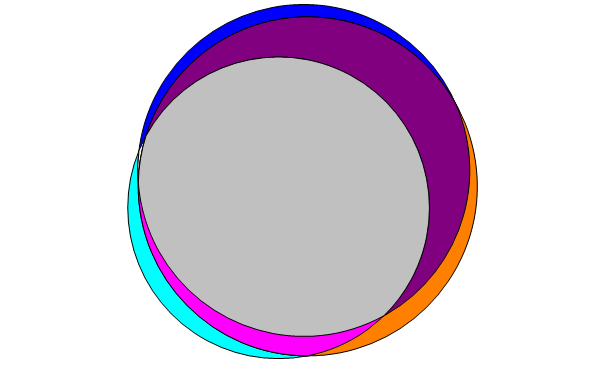


**17**

**1**

**1**

**1**

**1**

**5**


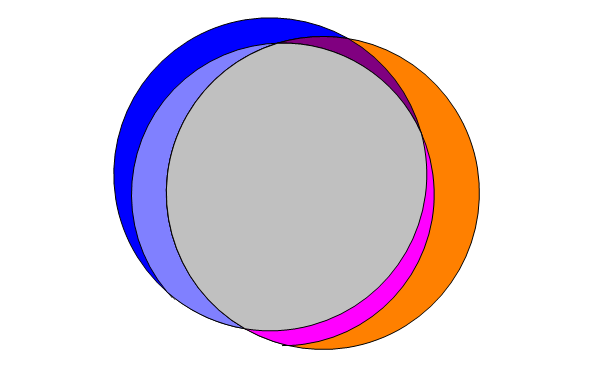


**23**

**4**

**1**

**2**

**5**

**3**

**S1 Figure:** Venn charts of pathway-associated proteins detected in P5, P10, and in adult (Ad) mice brain microvessels. (A) Distribution of proteins associated with energy metabolism: oxidative phosphorylation, citrate cycle (TCA cycle), glycolysis/gluconeogenesis, pyruvate metabolism, butanoate metabolism, propanoate metabolism, synthesis and degradation of ketone bodies, fatty acid metabolism, and pentose phosphate KEGG pathways. (B) Protein metabolism including ribosome, spliceosome, proteasome, phenylalanine metabolism, arginine and proline metabolism, tryptophan metabolism KEGG pathways. (C) Antioxidant mechanisms including peroxiredoxins, thioredoxins, and superoxide dismutase. (D) Transport including Slc, Abc, and Cav gene–coded proteins.
