# Supplementary material for: Proteomic and transcriptomic study of brain microvessels in neonatal and adult mice
Source: PLoS One. 2017 Jan 31;12(1):e0171048. doi: 10.1371/journal.pone.0171048 (PMC5283732; doi:10.1371/journal.pone.0171048)
Supplement: S2 File — Copy of the first part of the study [31]. (PDF) [file pone.0171048.s002.pdf]

Original Article

JCBFM

Journal of Cerebral Blood Flow & Metabolism
0(00) 1–19
© Author(s) 2016
Reprints and permissions:
sagepub.co.uk/journalsPermissions.nav
DOI: 10.1177/0271678X16630557
jcbfm.sagepub.com
SAGE

Major remodeling of brain microvessels during neonatal period in the mouse: A proteomic and transcriptomic study

Baptiste Porte[1], Julie Hardouin[2,3], Yasmine Zerdoumi[4], Céline Derambure[5], Michèle Hauchecorne[1], Nicolas Dupre[1], Antoine Obry[3], Thierry Lequerre[5], Soumeya Bekri[1,6], Bruno Gonzalez[1], Jean M Flaman[4], Stéphane Marret[1,7], Pascal Cosette[2,3] and Philippe Leroux[1]

Abstract

Preterm infants born before 29 gestation weeks incur major risk of subependymal/intracerebral/intraventricular hemorrhage. In mice, neonate brain endothelial cells are more prone than adult cells to secrete proteases under glutamate challenge, and invalidation of the Serpine 1 gene is accompanied by high brain hemorrhage risk up to five days after birth. We hypothesized that the structural and functional states of microvessels might account for age-dependent vulnerability in mice up to five days after birth and might represent a pertinent paradigm to approach the hemorrhage risk window observed in extreme preterms. Mass spectrometry proteome analyses of forebrain microvessels at days 5, 10 and in adult mice revealed 899 proteins and 36 enriched pathways. Microarray transcriptomic study identified 5873 genes undergoing at least two-fold change between ages and 93 enriched pathways. Both approaches pointed towards extracellular matrix, cell adhesion and junction pathways, indicating delayed microvascular strengthening after P5. Furthermore, glutamate receptors, proteases and their inhibitors exhibited convergent evolutions towards excitatory aminoacid sensitivity and low proteolytic control likely accounting for vascular vulnerability in P5 mice. Thus, age vascular specificities must be considered in future therapeutic interventions in preterms. Data are available on ProteomeXchange (identifier PXD001718) and NCBI Gene-Expression-Omnibus repository (identification GSE67870).

Keywords

Brain development, capillaries, pediatric stroke, mouse, proteomics, subependymal/intraparenchymal/intraventricular hemorrhage, transcriptomics

Received 18 September 2015; Revised 17 November 2015; 18 December 2015; Accepted 21 December 2015

Introduction

Various types of acquired neonatal brain lesions occur at specific ages and result from the co-incidence of deleterious environmental factors and specific stages of development.[1] In extreme preterms (less than 28

[1]INSERM-ERI28, NeoVasc Laboratory, Microvascular Endothelium and Neonate Brain Lesions, Normandie Université, Institute for Research and Innovation in Biomedicine (IRIB), Rouen, France
[2]UMR-6270, CNRS, Polymers, Biopolymers, Surfaces, Biofilm Resistance, Cell Surfaces Interactions Group (PBS), CNRS, IRIB, Normandie Université, Mont-Saint-Aignan, France
[3]Proteomic Facility PISSARO, IRIB, Normandie Université, Mont-Saint-Aignan, France
[4]UMR-S1079, INSERM, Genetic of Cancer and Neurogenetics (GCM), IRIB, Normandie Université, Rouen, France
[5]UMR-S905, INSERM, Pathophysiology and Biotherapy of Inflammatory and Autoimmune Diseases, IRIB, Normandie Université, Rouen, France
[6]Metabolic Biochemistry, Rouen University Hospital, Rouen, France
[7]Neonatal Pediatrics and Intensive Care, Rouen University Hospital, Rouen, France

Corresponding author:
Philippe Leroux, Normandie Université, NeoVasc Laboratory, Microvascular Endothelium and Neonate Brain Lesions, INSERM-ERI28, Faculty of Medicine and Pharmacy, Institute for Research and Innovation in Biomedicine (IRIB), 22 Bd Gambetta, 76183 Rouen, France.
Email: philippe.leroux@univ-rouen.fr

gestation weeks, the major risk is subependymal/intraventricular/intraparenchymal brain hemorrhage (SEH/IVH/IPH).[2] SEH/IVH/IPH affects extreme preterm infants in the periventricular germinal matrix revealing vascular vulnerability at this site during a specific period.[3] The germinal matrix is the site of intense metabolism and high oxygen consumption due to neural precursor cell multiplication, angiogenesis and microvessel remodeling with strict ontogenic schedule.[4,5] In addition, the germinal matrix is at risk of hypoperfusion due to fluctuation in cerebral blood flow in sick preterm infants.[6,7] Indeed, angiogenic factors exhibit pro-hemorrhage potential through extracellular protease activation.[3,8] The fact that endothelial cells (ECs) in rapidly growing neovessels have poor support from pericyte and astrocyte endfeets is another cause of vascular weakness.[9] Altogether, the actual vulnerability of vascular bed in the germinal matrix is multifactorial and clearly results from accumulation of hypoxia-ischemia/inflammation risk and structural weakness.

The blood to brain interface, referred to as the neurovascular unit, is the multicellular structure shaping ECs which regulate vascular permeability. The resulting blood–brain barrier (BBB) and blood to cerebrospinal fluid barriers restrain para-cellular diffusion and allow specific transendothelial transports. Although perivascular cell support is delayed, structural and functional studies have shown that microvessels have non-permeant and insult-resistant BBB early in development.[10,11] Expression of many transporter genes changes throughout development, reflecting changes in brain metabolism.[12] Indeed, a switch from monocarboxylate transporter1 (MCT-1) to glucose transporter 1 (GLUT-1) transporters was observed in cultured neonatal and adult brain microvascular ECs, in line with the view that energy metabolism in immature brain depends on monocarboxylates rather than on glucose.[13,14] Signaling at neonatal brain microvascular ECs also shows specificities. Neonate ECs express the N-methyl-D-aspartate (NMDA) receptor subunit NR1 and glutamate in these cells induces protease secretions (tissue plasminogen activator (t-PA) and gelatinases), whereas adult cells are not sensitive to the excitatory aminoacid.[15–17] The pro-hemorrhage potential of t-PA and the participation of gelatinases in vessel rupture are reported in adults as a consequence of the thrombolytic use of t-PA.[18,19] Several brain hemorrhage models in neonate and adult rodents are based on extracellular matrix (ECM) degradation based on local infusion of serine proteases, involved in coagulation and fibrinolysis[20] or matrix metalloproteinases (MMPs).[21,22] Gelatinases target collagens and BBB proteins,[23,24] while thrombin activates protease-activated receptors.[25] Complex interplay of serine proteases and MMPs also contributes to ECM degradation either by mutual cross-activation or through receptor-mediated inductions.[26–28]

In neonate mice invalidated on t-PA inhibitor-1 gene, age-dependent SEH/IVH/IPH could be mimicked up to five days after birth (P5) concomitantly with gelatinase activation within microvessels.[26,29] These observations support the fact that structural and functional specificities of brain microvasculature likely account for ontogenetic vulnerability window, and that mouse brain microvessels represent a heuristic paradigm for studying vascular immaturity as a favoring background for age-dependent neonate brain hemorrhage.

The present work was designed to describe brain microvessel protein content and transcription regulations around the period of vascular disruption propensity. Enriched fractions of mouse forebrain microvessels (fMVs) at three ages were prepared in the period of hemorrhage risk (P5), in immature although hemorrhage-resistant state (P10) and in adult (Ad) mice, then analyzed using proteomic and transcriptomic techniques and using gene ontology (GO) approaches.

Materials and methods

Animals

C57Bl/6 and Naval Medical Research Institute (NMRI) mice were bred in the housing facilities of the faculty of Medicine and Pharmacy of Normandy University and used according to the French law on ethics in experimental animal use (Articles R214-117 to R214-127 published on 7 February 2013) and the ARRIVE guidelines. Housing facilities authorization (B7645005) and protocol approvals (#01680.02) were given by the French Ministry of Higher Education and Research. Day of birth was considered day 1 of neonate life. Animals were kept at $21 \pm 1^{\circ}C$ with food and water *ad libitum* under a 7 a.m./7 p.m. light/dark cycle. Pups were sacrificed at P5, P10 or adulthood (P60 $\pm$ 5 days) for fMV extraction.

Fifty, 30 and 15 forebrains (both sexes) of C57Bl/6 mice were used to build up P5, P10 and adult protein samples, respectively. One hundred and twenty-five, 65 and 30 forebrains were needed to collect a minimum of 224 ng RNA, from P5, P10 and adult NMRI mice. Three independent fMV isolations were done for protein studies at P5 and P10, and two preparations for adult proteins and for each RNA extraction (Figure 1).

fMV extraction

fMVs were obtained as previously described.[17] Procedures were performed in RNAse-free conditions. Dissected brains were sectioned posterior to olfactory bulb and anterior to colliculi, freed of meninges in

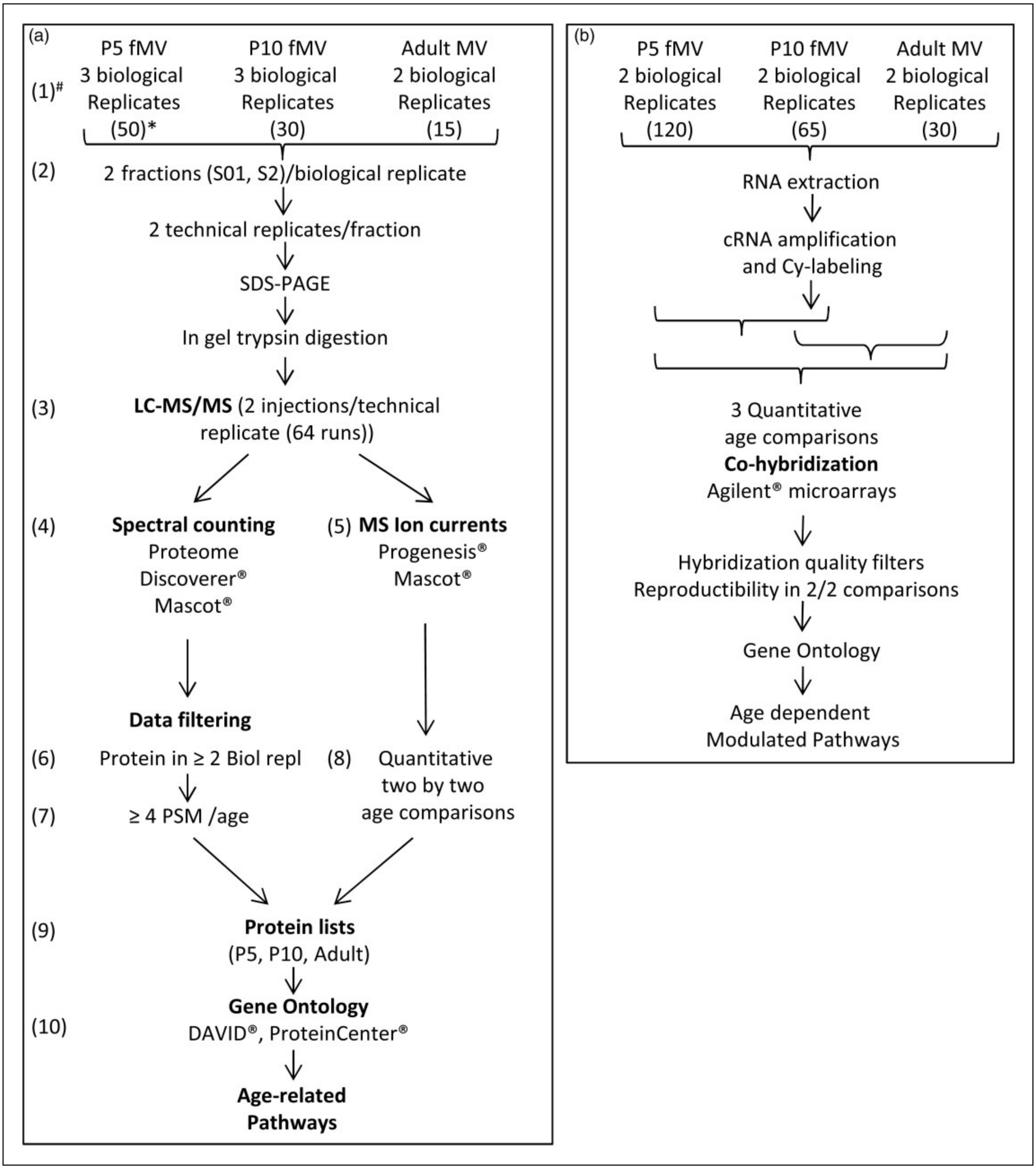

Figure 1. Experimental schedule. (a) Proteomic study, (b) Transcriptomic study. * indicates the number of pooled forebrains used for constitution of each biological replicate. # indicates steps of technical and bioinformatic analyses. Details are provided in the supplementary material file and supplementary Figure 2.

MCDB-131 (GE Healthcare, Velizy, France) supplemented with 2% fetal calf serum, 1% penicillin, 1% streptomycin and fongizol (complete MCDB). Tissue homogenization was performed in 7 mL complete MCDB using a Dounce homogenizer. The homogenate, suspended in 50 mL complete MCDB, was centrifuged (15 min, 1450 r/min, 4°C). The resulting pellet was resuspended in 50 mL complete MCDB containing 18% Dextran and centrifuged (30 min, 3200 r/min, 4°C). Supernatants obtained were centrifuged again to improve recovery yield. The two pellets, suspended in 5 mL complete MCDB, were

filtered on 70 μm mesh nylon membrane to discard debris. The filtrate was diluted in final 50 mL using complete MCDB, then centrifuged (7 min, 1650 r/min) and the pellet suspended in 200 μL sterile phosphate-buffered saline (PBS).

For the proteome study, each pellet was centrifuged again (7 min, 1650 r/min) in PBS, dried and frozen at −80°C until analysis.

Protein extraction

Dried fMV was suspended in 1 mL triethyl-ammonium bicarbonate buffer (0.2 M, pH 8.5; TEAB) and solubilized in three steps: (i) a mild sonication (65 W, 20 kHz, 1 min; Vibra-Cell, Sonic Material Inc., Newtown, CT) and centrifugation (15 min, 12,000 r/min, 4°C) provided the S0 fraction, (ii) a stronger sonication (104 W, 20 kHz, 1 min) of re-suspended pellet in 1 mL TEAB and centrifugation (15 min, 12,000 r/min, 4°C) provided the S1 fraction, (iii) the same strong sonication of previous pellet re-suspended in 1 mL in a highly denaturing buffer (urea 7 M, thiourea 2 M, tri(n-butyl) phosphine 0.05%, dithiothreitol (DTT) 20 mM, C7BzO 0.5%, CHAPS 2%, SDS 1%) allowed complete dissolution, providing fraction S2. Due to low protein concentration in S0 from P5 samples, S0 and S1 were merged (v/v) into a S01 combined fraction for each age. Protein concentrations were determined by Bradford technique.

Western blots were performed using antibodies against platelet endothelial cell adhesion molecule (PECAM) (sc-1506 1/1000, Santa Cruz Biotechnology, CA), platelet-derived growth factor receptors (PDGF-R) (sc90991 1/1000, Santa Cruz Biotechnology), Glial fibrillary acidic protein (GFAP) (sc-6170 1/1000, Santa Cruz Biotechnology) and synaptophysin (sc-52636 1/1000, Abcam, Paris, France) to validate fMV enrichment (See online supplementary material).

Tandem mass spectrometry

For each sample, 30 μg proteins were concentrated by gel electrophoresis (7% polyacrylamide, SDS; 0.1%) into a single band revealed by coomassie blue. Excised bands were treated as follows: In-gel proteins were reduced (DTT; 0.77 mg/mL, 10 min) and alkylated, (iodoacetamide; 1.85 mg/mL, 10 min), then submitted to trypsin digestion (0.1 μg/μL, 3 h). Resulting peptides were extracted from gel by successive washing with acidified acetonitrile water solutions. After drying, peptides were re-suspended in formic acid (FA; 0.1%) at a 0.2 μg/μL final concentration. Peptides (1 μg) were separated by nano liquid chromatography (Easy-nLC II, Thermo Fisher Scientific, Villebon/Yvette, France), first on enrichment column (Cap Trap C8, 0.5 × 2 mm, Michrom Bioresources, Auburn, CA) followed by a reversed-phase column (C18, L153, ID 5 μm, 100 Å pore size, Nikkyo Technos, Japan). The gradient (mobile phase A: H_2O/0.1% FA; mobile phase B: CH_3CN/0.1% FA) was delivered at 300 nL/min from 2 to 40% B in 105 min, then 40 to 80% B in 4 min and a final step at 80% B for 15 min. Eluted peptides from C18 were injected in LTQ-Orbitrap Elite (Thermo Fischer Scientific) mass spectrometer (MS) by electrospray ionization at 1.5 kV and 200°C. Tandem MS/MS was performed in a "data-dependent" mode. The 20 most intense ions in full scan MS were selected and fragmented (CID). MS and MS/MS analyses were done from 300 to 2000 m/z and scan resolution set at 60,000. The procedure of protein list elaboration is detailed in online supplementary Figure 2.

Transcriptome analyses

Pelleted fMV were suspended in lysis buffer with 1% ßmercaptoethanol and submitted to two runs of strong agitation (16 s at 50 Hz) with ceramic beads (1.4 mm, Ozyme, Montigny-le-Bretonneux, France) using a tissue lyser® (Qiagen, Courtaboeuf, France) for total cell disruption. Total RNA extraction was performed using the RNeasy Micro Kit® (Qiagen) and 260/280 integrity ratio above 1.9 and an RNA integrity number above 9.0 using a Nanodrop 2000 c spectrometer (Thermo-Scientific).

Two-color comparative hybridization was performed using Whole Mouse Genome Oligo 4 × 44 K Microarray (G4845A, Agilent Technologies, Les Ulis, France) to compare gene expression profiling in fMV from P5, P10 and adult mice. cRNA was synthesized from 100 ng total RNA and labeled using Quick Amp Labeling Kit (Agilent Technologies). A total of 825 ng of cRNA was co-hybridized on microarrays for 17 h at 65°C. Raw hybridization data evaluated on 5 μm pixel size using Agilent DNA microarray scanner G2565CA (Agilent Technologies) were extracted and normalized, then transferred to Genespring® (GX 12.6 software, Agilent Technologies) for data processing and data mining. All profiling of the three age comparisons was performed in two biological replicates. A "Dye Swap" assay made of reciprocally Cy3/Cy5 labeled P10 and adult RNA was included in each experiment (online supplementary Figure 1(b)). Data were in agreement with the Minimum Information on Microarray Experiment guidelines, deposited in the NCBI Gene Expression Omnibus (accession number: GSE67870).

In each array, outlier spots and those with heterogeneous signal on one color were discarded. Spots exhibiting a Cy3/Cy5 fold change ($|FC|$) >2 in the two experiments and $p \leq 0.05$ were selected for analysis, excluding spots with hybridization level <1st percentile

in the color or inter-array |FC| variation in each raw monochromatic signal >50%.

The Gene Ontology tool for GeneSpring® (Agilent technologies) allowed determination of significant statistical enrichment of biological processes based on computation p-values described by standard hypergeometric distribution. Differentially expressed genes were used for computational analysis to identify potential canonical and curated functional pathways using Wikipathways database imported in GeneSpring®. Gene expression profiling data were validated on some targeted genes by quantitative multiplex RT-PCR (Quantitative Multiplex polymerase chain reaction of Fluorescent Fragments).[30] A high correlation between |FC| was obtained between multiplexed PCR and hybridization arrays (online supplementary Figure 1(c)).

Bioinformatics, biostatistics and data management

A total of eight biological samples split in two fractions (S01–S2) treated separately in two technical replicates, each injected two times on LC-MS/MS provided 64 runs (Figure 1). Raw mass spectrometry files from LTQ-Orbitrap were analyzed separately by Proteome Discoverer® (v 1.3.0.339, Thermo Fisher Scientific) and Progenesis® LC-MS (v 4.0.4441.29989, Nonlinear Dynamics). Both software used Mascot® (v 2.2, Matrix Sciences) as identification engine according to IPI database (version 20070119). Identification parameters were: a cut-off identity score set at 25, tryptic peptides, one missed cleavage allowed, fragment mass tolerance set at 10 ppm, peptide tolerance set at 0.5 Da and took into account cysteine carbamidomethylation as fixed modification and methionine oxidation as variable modifications.

Proteome Discoverer® was used to describe protein profiles based on spectral counting (setting peptide confidence high). The criterion for protein recording at a definite stage of development was its occurrence after signal quality application in at least two independent biological samples at one stage (online supplementary Figure 2). Progenesis® LC-MS provided protein abundance inter-age comparisons, based on extracted ionic currents. Inter-age ANOVA statistic parameters were set at $p < 0.05$; $q < 0.05$ and power > 0.8 and applied at peptide and protein levels.

GO analyses were completed by GO annotation (cellular component, biological processes, metabolic functions) using Protein Center®, and the DAVID bioinformatics database freeware (version 6.7)[31,32] allowing Kyoto encyclopedia of genes and genomes (KEGG) pathway identification.[33,34] A Fischer exact test was used to assess KEGG pathways enrichment (threshold: $p < 0.05$). Protein lists were compared among samples, age and fractions using XL Comparator (online freeware, Marc Colson), Excel-2010 (Microsoft, Issy les Moulineaux) and Venn diagram online (online freeware, Microarray center, CRP-Santé, Luxembourg).

RNA samples from two independent experiments were hybridized on two distinct arrays for the three comparisons, i.e. P5–Cy5/P10–Cy3, P10–Cy5/Ad–Cy3, P5–Cy5/Ad–Cy3. Gene expression comparative analysis for developmental evolution profiles in P5 and adult fMV was conducted in a one-step study by co-hybridization of P5 and adult cDNAs (P5–adult period) and in a two-step study of P5–P10 and P10–adult comparisons. The three comparisons allowed discrimination of (i) early regulations (both senses) observed in the P5–P10 comparison, noted "early" whatever their following behavior, (ii) late regulations (both senses) observed in the P10–adult comparison and noted "late" and (iii) long-term evolutions observed in the P5–adult comparison. Slow evolutions referred to genes exhibiting P5–adult differences only. Few probes exhibited biphasic regulations. As mRNA samples were obtained from dozens to hundreds of pups, one may consider that the requirement of inter-assay reproducibility allowed statistically valuable conclusions. Intensity ratios were analyzed depending on variation sense along with age. A total of 6018 probes fulfilled these criteria in at least one age comparison coding 5873 genes. Invariant genes were determined on the same criteria except on FC set <0.05.

The Gene Ontology tool for GeneSpring® was applied on up- and down-regulated entities using the data of the two replicates of each age comparison.

Results

Protein study

Protein contents. Western blot identification of cell type markers of ECs and pericytes; PECAM and PDGF-R were present at all stages. Conversely, neonate fMV were largely free of GFAP and synaptophysin labeling astrocytes and neuron terminals, respectively (online supplementary Figure 1(a)). Retrospective research in protein lists from spectrometry reveal that astrocyte and neuron perikarya were absent in fMV.

A total of 899 proteins were identified in the study at the three age; 632 (70%), 727 (81%) and 692 (77%) proteins being identified in P5, P10 and adult fMV, respectively. Results from spectral counting analysis revealed 53% proteins common to the three ages (n = 476) (Figure 2(a)). Few proteins were age-specific (n = 29 (3%) at P5, n = 113 (13%) at P10 and n = 73 (8%) in adult fMV) or detected at two stages (P5/P10; n = 66 (7%), P10/adult; n = 82 (9%) and P5/adult; n = 61 (7%)). Transient proteins observed at P5 and/

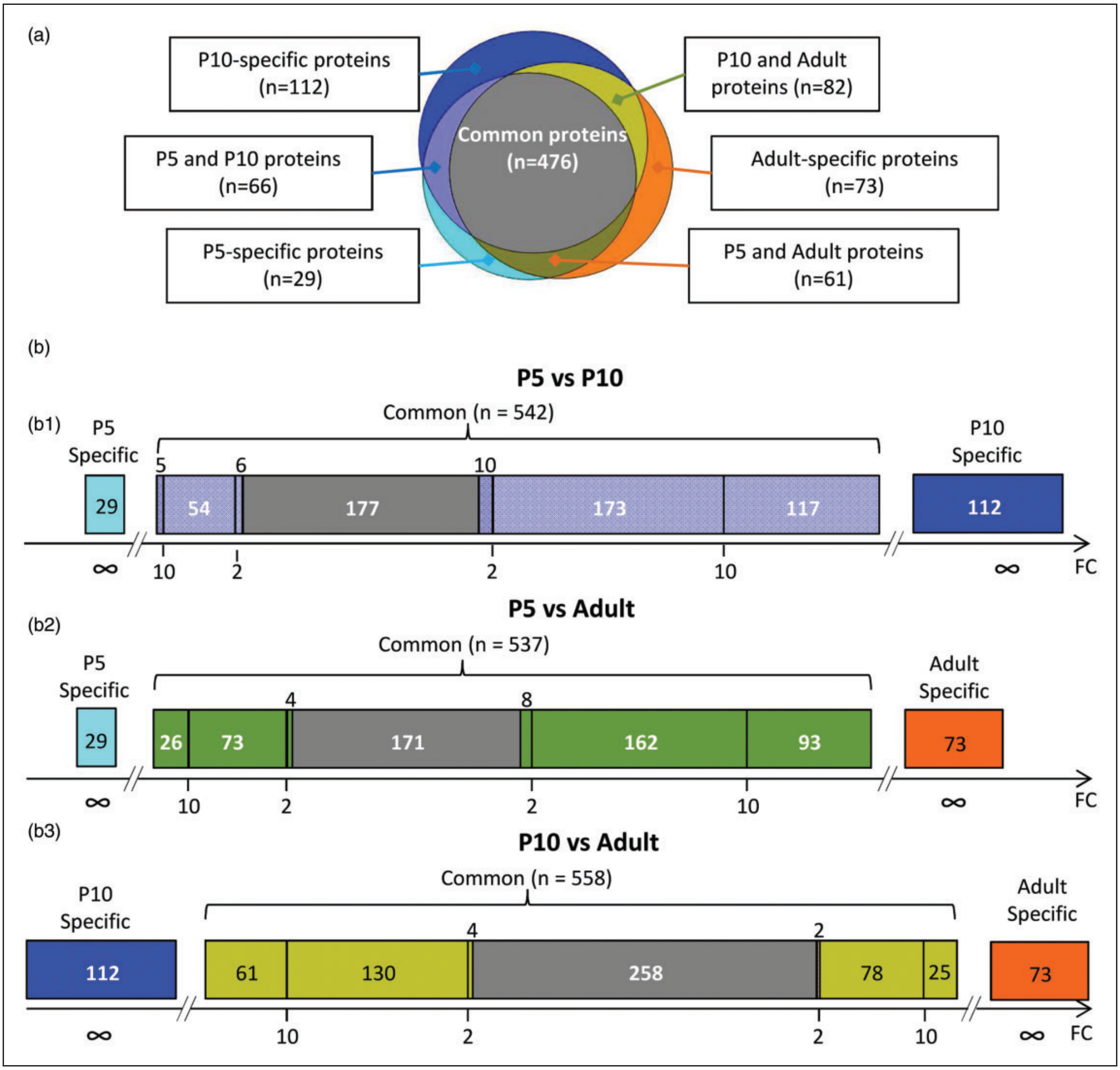

Figure 2. Comparative representation of protein occurrences, relative abundances observed in fMV from P5, P10, and adult mice. (a) Venn chart of distribution of 899 unique proteins in fMV at three ages. (b) Visualization of relative abundance for proteins in two by two age comparisons. B1: P5 to P10 integrating 29 proteins detected in P5 fMV only, 112 proteins detected in P10 fMV only and 542 proteins detected in both extracts and classified according to statistically significant differences (p-value < 0.05) in abundance and enrichment. B2: P5 to adult comparison; B3: P10 to adult comparison. Grey sectors indicate proteins with no difference. Light blue and dark blue were used for P5 and P10 stages; orange was used for adult proteins. Two- and 10-fold changes (FCs) are indicated in abscissa and highlighted by color. Color code in B refers to sectors in Venn plot in A.

or P10 were 23% of the total (n = 208). Reciprocally, proteins identified at P10 and/or in adults, considered proteins of maturity represented 17% of the total (n = 155). Thus, 72% of proteins were detected in at least two stages, but exhibited significantly different abundance (Figure 2(b)). P5 vs P10 and P5 vs adult comparisons exhibited very similar distributions with a minority of species enriched at P5 and 32–33% of species with non-significantly different abundances. Conversely, the P10 to adult comparison exhibited a high proportion of proteins enriched at P10 and 258 proteins (46%) with non-significantly different abundances (Figure 2(b)).

GO on proteome data. Protein lists annotated on cell localization, metabolic processes and molecular functions allowed segregation into GO categories. The Database for Annotation Visualization and Integrated

Discovery (David®) GO allowed identification of a total of 36 enriched KEGG pathways in P5, P10 and adult fMV. Seventeen were common to the three ages, mainly including basal metabolism, and structural or neurological disease associated pathways (online supplementary Table 1).

Protein contents in selected pathways related to vascular cohesion are provided in Table 1. In the pathway related to ECM-receptor interaction, seven of the eight collagen isoforms detected exhibited transient high levels at P10 (*Col1a2, Col4a2, Col6a1, Col6a2, Col6a3, Col12a1 and Col18a1*). Fibronectin exhibited the same profile (Table 1). Conversely, laminin isoforms showed late onset and were maximum in adults and demonstrated more than 10-fold enrichment between P5 and adult (*Lama2, Lama5, Lamc1*). CD47 antigen exhibited the same pattern (Table 1). These observations indicate profound remodeling of ECM during postnatal development. Indeed, the focal adhesion pathway including 21 proteins; laminins, collagens, fibronectin and beta-catenin showed statistically significant enrichment ($p = 0.023$) in adult fMV only.

Tight and gap junction pathways included 37 unique proteins, half of which were observed in fMV at the three ages. These pathways did not exhibit significant enrichment indicating an immaturity of BBB at P5 (Table 1, Figure 3(a)). Junction proteins Cldn11 showed a large increase after P10, while ZO-1 (Tjp1) and beta-catenin-1 (Ctnnb1) were maximal at P10 (Figure 3(a)). Among the nine tubulin isoforms detected and associated with gap junction pathway, alpha-4a and beta-4b isoforms appeared late, while isoforms beta-2b and beta-3 were highly reduced in adults. Few proteins putatively involved in transendothelial and efflux transports at the BBB were actually detected. Caveolines (1 and 2) were detected late and in highest amounts in adult fMV. The monocarboxylate transporters MCT-1 exhibited transient high at P10 while GLUT-1 was maximal in adult fMV. The neurotransmitter transporters EAAT-1, EAAT-2, GAT-1 also increased during development. But, the putative actors of glutamate transmission and excitotoxicity-induced extracellular proteolysis were not detected at the protein level, except serpinh1 (HSP47) which was present at all times.

Transcriptome analysis

Invariant genes ($FC < 0.05$ between two ages) were rare. No gene expression was invariant in all three age comparisons (Figure 4(a)). A total of 6018 probes fulfilled quality criteria and $FC > 2$ in at least one age comparison, coding 5873 genes. Nearly identical numbers of probes showed transcript up- (3197) or down-regulations (2821) (Figure 4(b) and (c)). Only 166 genes showed biphasic evolution. Dendrogram representation of the three age comparisons revealed high reproducibility between replicates and higher convergence between the P5 vs P10 and P5 vs adult comparisons than between one of the latter and the P10 vs adult comparison. The P10 vs adult comparison showed the highest number of invariants and the lowest number of up- or down-regulations, indicating that major changes occur before P10 (Figure 4(b) to (d)). Indeed, more than 40% of significant regulations appeared before P10 (up or down) and less than 25% were initiated after P10.

GO on transcript hybridization data. GO analysis detected 496, 429 and 121 significant GO features ($p < 0.05$) from early, late and slow probe lists, respectively. Most features were segregated in classes of functions associated with development, vessel development, metabolism regulation and signaling. Features related to membranes, extracellular components and cell adhesion appeared in early and late evolutions, but not in the P5 to adult slow evolution period.

Ninety-three enriched pathways were identified (Figure 4(e)). Online supplementary Table 2 reports age-comparison-enriched pathways, statistical significance and numbers of up- and down-regulated genes in each pathway. Eighteen pathways were detected in the three age comparisons, but less than 8% of genes in these pathways exhibited $FC > 2$ in at least two periods.

Fifty-five enriched pathways were identified from early regulated genes. Five pathways were related to development including cell cycle features and apoptosis. A high proportion was related to signaling at intercellular level (TGF, EGF, insulin, GPCR, androgens) intracellular level (Wnt, ErbB, G-protein synaptic proteins, MAP kinases) or nuclear level (Notch, Hedgehog, folic acid), including immune and inflammation signaling (IL1, IL4, TLR, T and B-cell receptor signaling, complement). Six pathways were related to cell adhesion, cytoskeleton and motricity (online supplementary Table 2.1). These pathways contained the highest number of genes, i.e. fibronectin and vitronectin exhibiting high expression levels, and extracellular proteins.

Fifty-five enriched pathways were identified from late regulated genes. In this P10–adult period, pathways related to development (cell cycle and apoptosis) mainly exhibited decreasing gene expression (online supplementary Table 2.2). Many pathways also highlighted signaling at extracellular level (insulin, TGF, HGF, EGF, chemokines, SIP signaling), intracellular level (Wnt, ErbB, G-proteins synaptic proteins, MAP kinases), nuclear level (nuclear receptors, androgen receptors) or immune/inflammation systems (IL3, IL5, IL7, B and T-cell receptor signaling, γ-interferon).

Table 1. Proteins identified in P5, P10 and adult fMV and quantified using ionic current intensities and/or spectral counting.

UNIPROT	GENE	Ionic currents[a] ($\times 10^{-3}$) Cumulated normalized abundance Progenesis® analysis			PSM[b] Proteome discoverer® analysis		
		P5	P10	Adult	P5	P10	Adult
**ECM-receptor interaction pathway**							
P02468	*Lamc1*	1.3 ± 0.4[c]	7.9 ± 3.9	36.4 ± 14.5	–	15	51
P08122	*Col4a2*	3.0 ± 0.9	151[d]	84.8	–	16	12
P11087	*Col1a1*	–	–	–	–	6	9
P11276	*Fn1*	1.0	8.6 ± 1.3	1.4	–	16	–
P97927	*Lama4*	–	–	–	–	9	
Q01149	*Col1a2*	0.3	5.9 ± 0	0.2	–	9	–
Q02788	*Col6a2*	–	–	–	–	5	–
Q04857	*Col6a1*	0.4	9.9 ± 2.9	0.4	–	25	–
Q60675	*Lama2*	1.1 ± 0.3	1.7 ± 1.3	32.7 ± 11.8	–	–	10
Q61001	*Lama5*	2.7 ± 1.4	14.4 ± 6.0	106 ± 45	–	–	81
Q61292	*Lamb2*	5.7	12.4 ± 11.2	19.4	–	25	76
Q61735	*Cd47*	8.9	15.8	70.4	13	15	3
**Focal adhesion pathway**							
O35558	*apk1*	–	–	–	–	4	–
O88643	*ak1*	–	–	–	–	–	6
P02468	*Lamc1*	1.3 ± 0.4	7.8 ± 3.8	36.4 ± 14.5	–	15	51
P08122	*Col4a2*	3.0 ± 0.9	151	84.8	–	16	12
P11087	*Col1a1*	–	–	–	–	6	9
P11276	*Fn1*	1.0	8.6 ± 1.3	1.4	–	16	–
P49817	*Cav1*	7.4 ± 0.0	45.8	65.2	–	1	4
P57780	*Actn4*	0.1	1.6	–	–	27	14
P60710	*Actb*	878 ± 58	792 ± 337	185 ± 19	136	180	160
P62835	*Rap1a*	3.8 ± 1.1	25.9	16.7	–	–	–
P97927	*Lama4*	–	–	–	–	–	9

(continued)

Table 1. Continued

		Ionic currents[a] ($\times 10^{-3}$) Cumulated normalized abundance Progenesis® analysis			PSM[b] Proteome discoverer® analysis		
UNIPROT	GENE	P5	P10	Adult	P5	P10	Adult
Q01149	*Col1a2*	0.3	5.9 ± 0.0	0.2	–	9	–
Q02248	*Ctnnb1*	–	8.7	0.9	–	9	–
Q02788	*Col6a2*	–	–	–	–	5	–
Q04857	*Col6a1*	0.4	9.9 ± 2.9	0.4	–	25	–
Q60675	*Lama2*	1.1 ± 0.3	1.7 ± 1.3	32.7 ± 11.8	–	–	10
Q61001	*Lama5*	2.7 ± 1.5	14.4 ± 6.0	106 ± 45	–	–	81
Q61292	*Lamb2*	5.7	12.4 ± 11.2	19.4	–	25	76
Q63844	*Mapk3*	–	1.2	0.1	–	–	–
Q6ZWQ9	*Myl12a*	25.5	80.0	–	14	35	31
Q8BTM8	*Flna*	0.5	15.9 ± 0.9	1.4	–	–	–
Q9CQ19	*Myl9*	1.9	–	25.4	–	19	21
Q9WVC3	*Cav2*	–	–	–	–	–	6
**Tight junction and gap junction pathways**							
2RSH2	*Gnai1*	–	7	26.3	–	7	16
O35558	*Mapk1*	–	–	–	–	4	–
P08752	*Gnai2*	10.3	–	42.2	–	12	–
P10833	*Rras*	–	–	–	–	–	9
P11440	*Cdk1*	–	–	–	4	7	–
P21278	*Gna11*	–	0.4	0.1	–	–	–
P21279	*Gnaq*	7.2 ± 1.1	19.0	23.9	6	6	16
P39447	*Tjp1*	0.4	10.0	–	–	17	8
P57780	*Actn4*	0.1	1.6	–	–	27	14
P60710	*Actb*	878 ± 59	793 ± 337	186 ± 19	136	180	160
P62071	*Rras2*	1	–	5.5	–	12	15
P63054	*Pcp4*	0.1	–	–	–	–	7
P63094	*Gnas*	–	–	–	–	6	–
P68181	*Prkacb*	–	–	–	–	–	6
P68368	*Tuba4a*	–	–	–	–	73	82
P68369	*Tuba1a*	909 ± 169	609 ± 179	114 ± 72	111	91	76
P68372	*Tubb4b*	16.9	22.8	61 ± 5	–	172	93

(continued)

Table 1. Continued

UNIPROT	GENE	Ionic currents[a] ($\times 10^{-3}$) Cumulated normalized abundance Progenesis® analysis			PSM[b] Proteome discoverer® analysis		
		P5	P10	Adult	P5	P10	Adult
P68373	*Tuba1c*	–	–	74	32	–	
P99024	*Tubb5*	136.0	–	143.0	137	246	64
Q02248	*Ctnnb1*	–	8.7	0.9	–	9	–
Q60737	*Csnk2a1*	–	–	–	–	5	–
Q60771	*Cldn11*	6.8	17.6 241 ± 23	–	–	15	
Q61879	*Myh10*	9.2 ± 9.2	2.0	4.0	20	50	–
Q62261	*Sptbn1*	73.0 ± 70.9	18.0	82.9 ± 70.7	53	127	158
Q63844	*Mapk3*	–	1.2	–	–	–	–
Q6ZWQ9	*Myl12a*	25.5	83.3	–	14	35	31
Q76MZ3	*Ppp2r1a*	9.6 ± 0.0	6.7 ± 5.6	3.3 ± 1.5	8	8	–
Q7TMM9	*Tubb2a*	11.4	96.4	–	132	276	169
Q7TMQ1	*Gja1*	–	–	–	–	4	–
Q8VDD5	*Myh9*	0.5	3.2	–	–	9	–
Q8VED4	*Ppp2cb*	–	–	–	–	–	18
Q9CQ19	*Myl9*	1.9	–	25.4	–	19	21
Q9CWF2	*Tubb2b*	83.9 ± 12.7	27.4	12.0	132	253	–
Q9D6F9	*Tubb4a*	–	5.5	22.7	68	102	87
Q9ERD7	*Tubb3*	9.1	–	72.3	89	139	–
Q9JKB3	*Ybx3*	–	–	–	–	11	–
Q9WV92	*Epb41l3*	0.7	–	15.6	–	–	13

PSM: peptide spectrum match. [a]Filters set as, ANOVA $p < 0.05$. $q < 0.05$, power > 0.8 at peptide and protein analysis levels. [b]Quality filter sets on high peptide confidence. [c]Mean ± SEM of cumulated normalized abundance in two age comparison. [d]Cumulated normalized abundance calculated at one age measured in only one comparison.

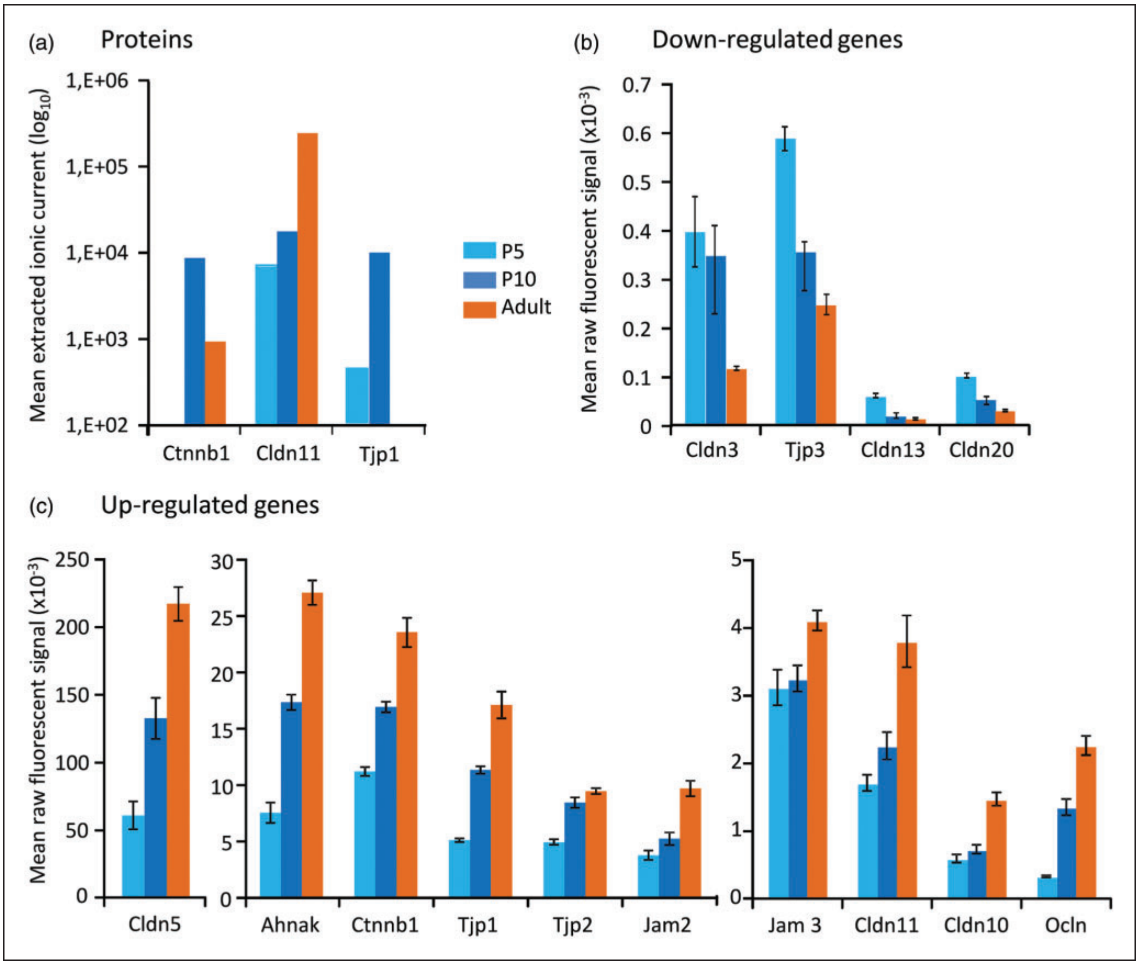

Figure 3. Tight and gap junction protein contents and RNA hybridization signals. (a) Proteins, (b) genes exhibiting age-dependent expression decrease, (c) genes exhibiting age-dependent expression increase.

Cell adhesion-related pathways showed high enrichment and thus low p values.

Forty-three pathways were extracted from long-term regulated genes (online supplementary Table 2.3). The majority concerned signaling at extracellular level (EGF, insulin, HGF, TGFb, chemokine, TLR and GPCR including S1P signaling), intracellular level (MAP kinases, Notch and PPAR), nuclear level (nuclear receptors) and immune/inflammation systems (IL1, IL2, IL3 and T-cell receptors). Cell adhesion-related pathways were also observed, although at lower statistical significance than in P10-centered periods.

Since no recorded pathway described the mechanisms involved in brain microvascular physiopathology (remodelling, basal lamina, BBB, excitotoxicity), we investigated self-made gene/protein lists (Table 2, online supplementary Table 3). Factors putatively involved in microvessel vulnerability (ECM components, glutamate receptors, proteases and inhibitors) exhibited age-dependent evolution (online supplementary Table 3.1, 3.2 and 3.3, respectively). Many genes coding collagen isoforms exhibited up-regulation between P5 and P10, then expression decreased. Similar kinetics was observed for the major collagen isoforms at protein level (*Col1a1, Col1a2, Col3a1, Col4a1, Col18a1 or Col26a1*). Laminin gene transcription levels displayed either transient highs at P10 (*Lama1*, *Lama2*, Lama4, *Lamb1* and *Lamc1* isoforms) or expression increases from P5 to P10, and continuous rise to adult (*Lama5*, *Lamb2*) (online supplementary Table 3.1). Several proteins exhibited coherent levels (*Lama2, Lama5*, *Lamb2, Lamc1*) (Table 2). ECM interaction molecule genes such as genes coding fibronectin, vitronectin and integrin showed a variety of age-dependent patterns (online supplementary Table 3.1). At protein level, the sole integrin-associated protein (CD47) was detected showing late increase (Table 1). Relative to BBB, most tight junction protein coding genes exhibited progressive age-dependent rise: *Cldn5*, *Ctnnb1*, *Tjp1*, *Tjp2*, *Jam2*, *Jam3*, *Cldn11*, *Cldn10* and *Ocln* (Figure 3(c)). Some genes underwent progressive decrease, although at low expression level; *Tjp3*,

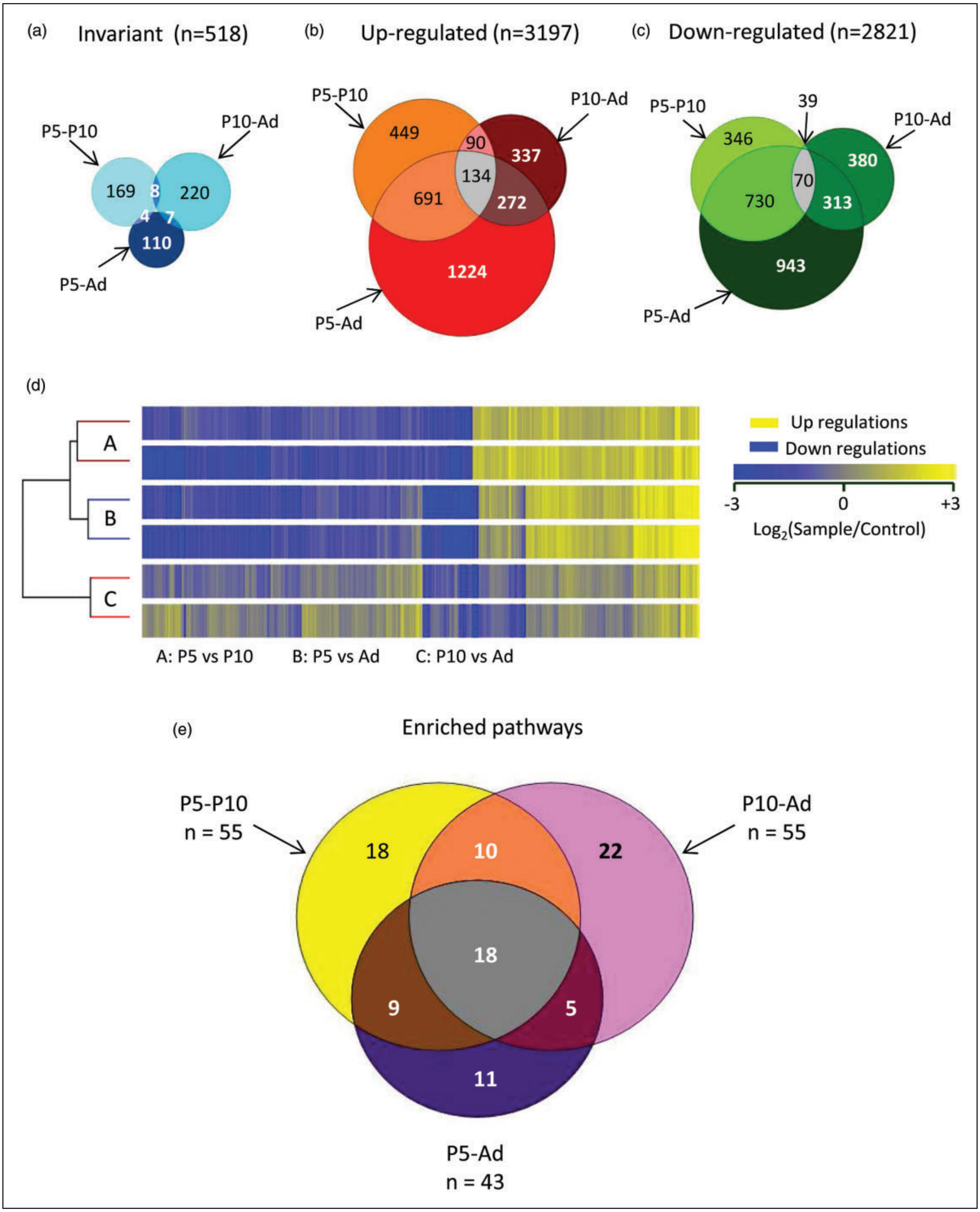

Figure 4. Distribution of gene expression regulations over the three age comparisons. (a) Venn chart of the 518 genes observed with invariant expression ($FC < 0.05$) in at least one comparison. (b) Venn chart of the 3197 up-regulated genes ($FC > 2$). (c) Venn chart of the 2821 down-regulated genes ($|FC| > 2$). (d) Dendrogram of two independent replications of three comparative expression experiments. Each ray corresponds to one gene over the 6018 exhibiting a $FC > 2$ in the two replicates in at least one age comparison. Maximum color intensity was set at $FC \geq 3$. (e) Venn chart distribution of the 93 enriched pathways in the three age comparisons.

Table 2. Transcript levels of major components of basal lamina and adhesion molecules.

Genes	P5	P10	Adult
Col1a1	8.34 ± 0.70[a]	**14.80 ± 1.29**	2.52 ± 0.21
Col1a2	41.97 ± 6.52	**65.71 ± 2.74**	17.03 ± 0.62
Col3a1	7.80 ± 1.00	**16.23 ± 1.11**	2.02 ± 0.05
Col4a1	15.19 ± 2.40	**43.35 ± 4.20**	10.88 ± 0.46
Col4a2	12.80 ± 0.92	**32.11 ± 1.88**	10.46 ± 0.68
Col4a5	1.11 ± 0.12	**2.14 ± 0.09**	0.69 ± 0.06
Col6a2	0.59 ± 0.06	**1.18 ± 0.16**	0.24 ± 0.01
Col6a3	0.98 ± 0.10	**1.70 ± 0.16**	0.28 ± 0.02
Col7a1	6.00 ± 0.32	**6.37 ± 0.44**	1.54 ± 0.19
Col8a1	0.34 ± 0.03	**1.00 ± 0.10**	0.39 ± 0.16
Col9a2	**2.25 ± 0.12**	1.37 ± 0.09	0.51 ± 0.04
Col16a1	**9.38 ± 0.17**	6.26 ± 0.52	3.28 ± 0.21
Col 18a1	8.01 ± 0.65	**34.12 ± 1.66**	5.59 ± 0.37
Col26a1	8.19 ± 0.35	**11.60 ± 1.60**	1.08 ± 0.08
Lama1	10.51 ± 1.26	**26.07 ± 0.23**	5.88 ± 0.39
Lama2	2.81 ± 0.42	**6.76 ± 0.87**	5.22 ± 0.35
Lama3	0.17 ± 0.02	**0.42 ± 0.02**	0.35 ± 0.03
Lama4	4.87 ± 0.27	**9.89 ± 0.83**	3.44 ± 0.19
Lama5	3.66 ± 0.13	9.06 ± 1.26	**11.89 ± 0.82**
Lamb1	14.70 ± 1.52	**24.84 ± 2.02**	4.29 ± 0.19
Lamb2	4.87 ± 0.64	12.08 ± 1.12	**13.97 ± 1.11**
Lamc1	9.83 ± 0.62	**22.60 ± 0.39**	11.16 ± 0.72
Itgam	**68.02 ± 1.87**	31.29 ± 2.34	34.19 ± 4.60
Itg6	12.79 ± 0.44	18.14 ± 1.61	**51.08 ± 4.61**
Itg4	3.07 ± 0.19	**6.09 ± 0.54**	1.66 ± 0.04
Itga1	1.59 ± 0.15	**5.41 ± 0.51**	4.61 ± 0.13
Itgb1	3.31 ± 0.16	**7.58 ± 0.49**	4.49 ± 0.28
Itg10	1.87 ± 0.08	**3.40 ± 0.45**	1.32 ± 0.12
Itg11	1.26 ± 0.09	**1.80 ± 0.25**	0.44 ± 0.01
Itga8	0.46 ± 0.04	**1.12 ± 0.08**	0.98 ± 0.04
Fn1	33.87 ± 0.32	**118.04 ± 8.65**	88.00 ± 6.94
Vtn	22.69 ± 1.73	51.90 ± 2.74	**127.46 ± 9.55**
CD47	4.31 ± 0.39	4.09 ± 0.32	**6.10 ± 0.11**

[a]Mean values ($\times 10^{-3}$) of four raw fluorescence determinations (two replicates and two age comparisons. Maximum values are in bold.

Cldn3, *Cldn13* and *Cldn20* (Figure 3(b)). Tight and gap junction-related proteins are detailed in Table 1 and mRNA regulations in online supplementary Table 3.2.

Ionotropic glutamate receptors were not detected at protein level. mRNA expression mainly exhibited decreasing evolution pattern (Figure 5(a), online supplementary Table 3.3). The only increases were *Grin2a* and *Grin2c*. The common NMDA receptor subunit *Grin1* was invariant while *Grin2b* and *Grin3a* decreased. The *Grin2d* and *Grin3b* isoforms were near detection limit. α-amino-3-hydroxy-5-methyl-4-isoxazolepropionic acid (AMPA) receptor *Gria1* and *Gria3* did not show high variations, while the common AMPA isoform *Gria2* and the *Gria4* coding genes underwent major reduction from P5 to P10. The same pattern was observed for all five kainate receptor isoform coding genes *Grik1-5*. Among metabotropic glutamate receptors, the only variations observed were a drop in *Grm5* and *Grm7* coding genes between P5 and P10. On the whole, these evolutions sustained high glutamate sensitivity in P5 neonates followed by quick regulation from P10 onwards.

Very few proteases and inhibitors were observed as proteins at all stages, i.e. several constant proteasome complex elements and serpina1d; or at specific time points; Caspase 3, Cathepsin H and sepinh1 in P5, P10 or adult, respectively. Conversely, at transcription level, significant two-fold variation changes were observed for many proteases (n = 37) and inhibitors (n = 33) (Figure 5 (b) and (c), online supplementary Table 3.4). mRNA regulation of protease-coding genes affected essentially zinc proteases (MMP, A Disintegrin and Metalloproteinase (ADAM), A Disintegrin And Metalloproteinase with Thrombospondyn Motifs (ADAMTS) and ADAMTS-like proteins (Adamtsl)), cysteine proteases (Caspases, Capn12) and only two serine protease genes (Htra1 and Plau). Zinc proteases have extracellular activities including extracellular zymogen maturation. Serine proteases also participate in initiation of Zinc-protease activation cascades. Ontology describes mainly ECM and adhesion functions, and to a lesser extent apoptosis and cytoskeleton functions. While similar numbers of protease coding genes exhibited age-related up- and down-regulations, suggesting age-dependent changes in actors of extracellular modeling, 27 of the 33 protease inhibitor genes showing significant postnatal evolution exhibited age-related up-regulations (Figure 5 (b) and (c); online supplementary Table 3.4). Zinc proteases predominate but inhibitor modulation mainly affected serine-protease inhibitors (26 genes), cysteine-protease inhibitor (*Cast*) and only two zinc-protease inhibitor genes (Timps). *Timp1* and *Timp3* exhibited high expression levels and late (P10–adult period) antiparallel evolution. Calpain12 and its inhibitor calpastatin genes exhibited the same early onset after P5.

These observations suggest that an extracellular proteolytic tone of serine proteases may prevail in neonates, which could be progressively blunted by late onset expression of inhibitors.

Proteome and transcriptome convergences

Seventy percent of the proteins identified (n = 631) did not exhibit 2-FC gene regulation. The remaining 30% showed |FC|>2 positive (n = 138) or negative (n = 130)

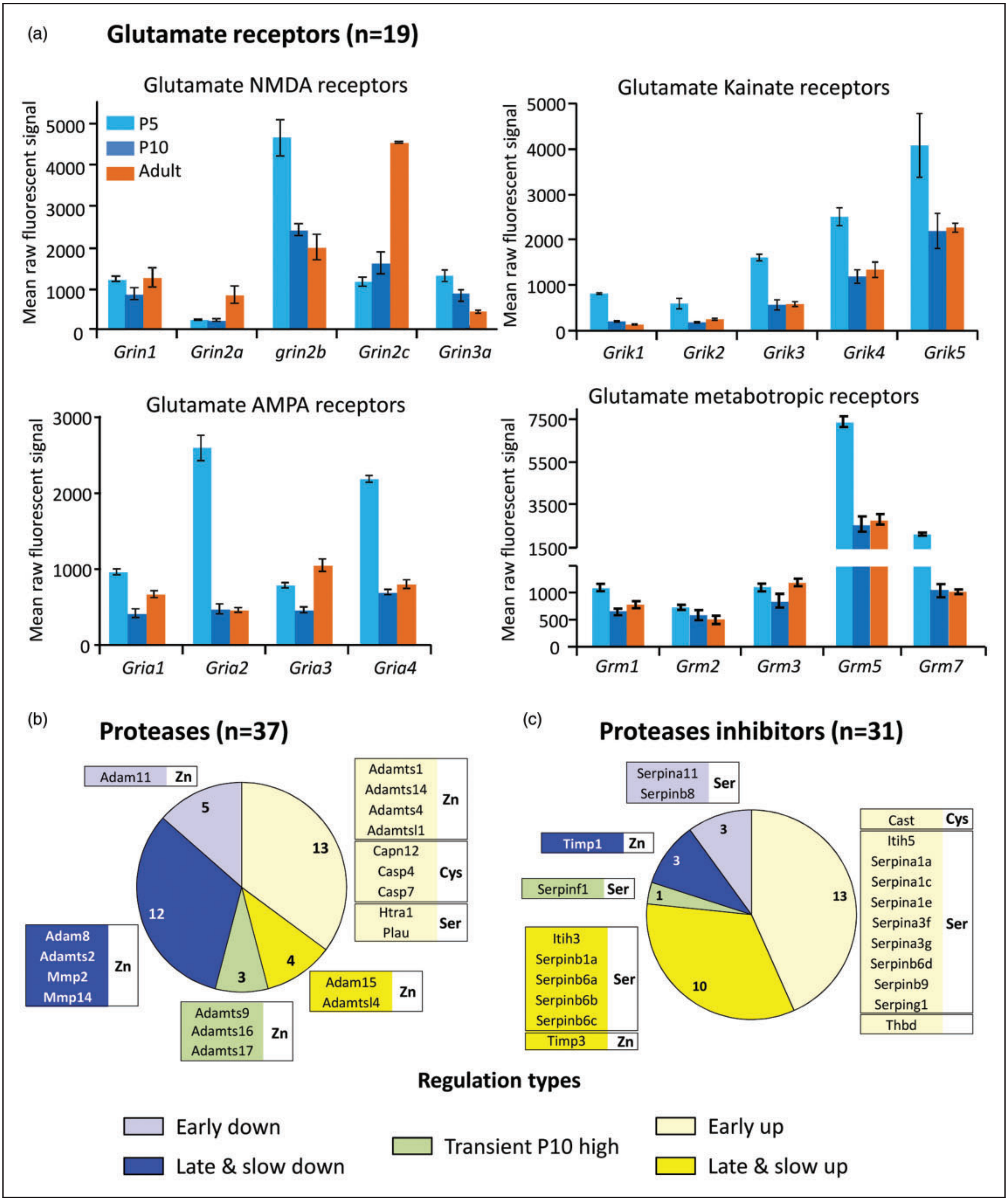

Figure 5. Transcription developmental patterns of glutamate receptor subtypes, proteases and protease inhibitors genes. (a) Expression levels (raw fluorescence) of 19 glutamate receptor subunits at the three ages. Values represent mean (±SEM) of 4 determinations. (b) Distribution of 37 significantly regulated genes coding proteases in 5 color-coded developmental patterns. n in sectors indicate the total number of genes in the group. Boxes recapitulate top 50% highest expression genes in each group. Zn, Cys, and Ser indicate Zinc, Cysteine and serine protease families, respectively. (c) Distribution of 31 significantly regulated genes coding protease inhibitors in five color-coded developmental patterns. Sectors and boxes are organized as in A. Zn, Cys and Ser refers to respective families of enzymes inhibited.

variations in one time interval. These genes encoded proteins observed at the three ages (59%), transiently (23%), or late (18%) (online supplementary Table 4). Although the two approaches differed in sensitivity, they converged towards common pathways. The GO analysis restricted to the 268 entities observed under both protein or mRNA regulation allowed identification of 11 significantly enriched pathways, of which focal adhesion, ECM-receptor interaction, gap junctions and tight junction pathways exhibited the highest number of entities and the highest statistical significance ($p < 0.001$). Further analyses on entity lists including only up or only down regulated entities even allowed detection of enrichment in focal adhesion, and ECM-receptor interaction pathways. Conversely, while tight junction pathway enrichment was noted in up-regulated or permanent entities, gap junction pathway appeared only associated with down-regulated and transient entity lists. In parallel, regulation of actin cytoskeleton, vascular smooth muscle contraction and leucocyte trans-endothelial migration pathways appeared significantly enriched ($p < 0.01$) in late- and up-regulated entity lists (online supplementary Table 4.4).

Discussion

The aim of the present work was to document as exhaustively as possible, and without *a priori* towards pathways or chemical families, the chemical composition and contents of mouse fMV as a model of immature vessels that break up in SEH/IVH/IPH. The study was performed in mice at P5, a period at risk of bleeding mimicking some aspects of SEH/IVH/IPH observed in extreme preterms due to vulnerability of vessels in germinal matrix and white matter; at P10, a later immature stage with decreased vulnerability in these structures as observed in at-term human newborns[29] and in adults. The deliverables expected were identification of structural and/or functional features of maturity, putative sources of vulnerability or resistance to environmental hazards.

The use of fully solubilized fMV rather than subcellular fractions was imposed by age-dependent differences regarding the sensitivity of fMV to mechanical and detergent actions that would have led to heterogeneous intermediate yields throughout the procedure. Indeed, our broad spectrum analysis was impeded by a loss of sensitivity compared to data in the literature on MS approaches in enriched fractions.[35] Although a wider range of fMV protein was used, it allowed detection of a high proportion of ECs (77%) and pericyte (47%) markers identified in an adult membrane MS study.[35] But, besides this limitation, analysis of pathways appeared more pertinent than study of subcellular fractions, since it included actors from extracellular to cell nuclear levels. In addition, separate GO extraction of enriched pathways allows comparison of protein and mRNA data with distinct sensitivity and constraints; protein occurrences were detected on the basis of the highest abundance at one age and dynamic mRNA comparisons recorded on variation of their expression regardless of basal level. The extreme sensitivity of transcriptomic study indeed provided more factors than proteomic study, e.g. glutamate receptors were far less abundant than ECM components. Analysis at the integrated level of pathways from the two sets of data in fact demonstrated convergences and allowed us to focus on specific proteins. Moreover, pathway determination performed on the few entities detected as both protein and RNA expression modulation focused to the highest significance on the same pathways: focal adhesion, ECM, tight and gap junctions.

Indeed, pathways related to growth factors, angiogenic factors and development appeared significantly enriched at transcription level in the comparisons including young stages. However, the corresponding proteins present in far lower amounts than structural proteins were hardly detected. The proteins observed refer to structural elements: basal metabolism, ECM, adhesion and transport functions. Few junction proteins of BBB were detected, but the data are compatible with the concept of a functional BBB early in development, and supported by early detection of transcripts, and by the BBB marker AHNAK.[36,37] Some junction protein transcripts also decreased, although their expression levels were low, supporting the notion of BBB element replacement during development, as previously reported regarding other BBB actors, e.g. transporters.[12,17]

Proteome analysis allowed determination of 899 proteins and 36 pathways reporting major activities in fMV during postnatal development. Dynamic analysis of gene transcripts underlined factors related to vascular cohesion. ECM, intercellular junctions and adhesion factors to basal lamina were the common pathways observed throughout the period. Nevertheless, constituents of these pathways demonstrated either substitution or quantitative change in molecular species from P5 to adulthood. These structural differences in ECM, EC adhesion components and transporters (data not shown) within fMV, likely underlie putative differences in physiopathology between P5 and P10 or adult mice, including BBB functions and mechanical resistance.

Vascular weakness, erratic hemodynamics and coagulation deficiency are common hypotheses evoked to explain high occurrence of SEH/IVH/IPH in early preterms. The biochemical mechanisms of vascular rupture remain less investigated.[3,7,9,38] Vascular endothelial growth factor (VEGF) has essential time-dependent function in cerebral vessel maturation and experimental blockade at definite periods could evoke an ultimate

pattern of periventricular leucomalacia.[5] VEGF also has deleterious potential as it promotes endothelium sprouting contributing to weakening of ECM. These effects involve MMP (ADAM) secretions.[8,39]

Few studies have reported brain vascular proteome data, and most focus on BBB function after insults,[40] or are based on cultured brain microvascular ECs.[41,42] This present study focuses on the physiological patterns of at-risk stages for bleeding and constitutive elements involved in fMV cohesion. Pathways related to focal adhesion, ECM, integrin-mediated cell adhesion and integrin signaling cumulate gene coding factors responsible for EC adhesion to each other or to basal lamina. At transcript level, two-thirds of ECM and adhesion protein-coding genes exhibited age-dependent increased expression, and several of them showed significant transient maximum at P10, supporting the hypothesis of microvascular weakness at P5, and the strengthening already observed at P10. The antiparallel decrease in collagens and increase in laminins indicate qualitative remodeling of ECM in this period of mouse postnatal development. The substitution of many collagen isoforms present at P5 by laminin isoforms observed in adults is not conclusive, regarding vascular weakness, but the concomitant increase in adhesion molecules (integrins, Fbn, Vtn, CD47) and junction molecules (claudins) sustains the idea of progressive strengthening of the intercellular and ECs to substrate junctions.

t-PA is an inducer of MMP cascades in adult brain microvessels, leading to BBB permeabilization, edema or vascular rupture.[43–45] The bleeding pattern restricted to the first five postnatal days in mice with t-PA inhibitor-1 gene knockout (Serpine1$^{-/-}$) indicates that both proteolytic activities and respective substrates are the elements of vulnerability.[29] The absence of major modulation of t-PA or Serpine1 gene expression does not exclude their participation in deleterious cascades in neonates but precludes their involvement as factors of age dependence. The central role of vascular basal lamina is highlighted by the dramatic neonatal hemorrhages accompanying *Col4a1* mutations in humans and mimicked in genetically engineered mice.[46,47] Also, vessel to parenchyma adhesion is disrupted in *Itga5* knockout mice and leads to brain *in utero* bleeding.[48] Of note, *Col4a2* protein exhibited a 50-fold increase between P5 and P10, and mRNA coding *Col4a2* or *Itga5* exhibited maximum at P10.

The age-dependent rise in expression of protease inhibitors is in line with the concept of active proteolytic degradation of perivascular matrix during early stage angiogenesis followed by a late decrease. No clear correlation between proteases and their inhibitors appeared in zinc or Serine protease families. Although Serpins show high diversity, it should be keep in mind that only four Timps inhibit the broad spectrum of zinc proteases with low selectivity. How these enzymes degrade extracellular environment or EC adhesion at early stages requires investigation at protein and activity levels. A suspected low proteolytic inhibitory tone in neonates may sustain a deleterious potential in microvessels exposed to environment insults. A relationship between glutamate and protease secretions in age-dependent microvessel vulnerability is supported by many observations. Glutamate uptake is low in neonates,[49] and expression of most glutamate receptor subtypes decreases with age (present data). Glutamate evoked t-PA and gelatinase secretions by neonate but not adult brain microvascular ECs in culture[17] and had a higher potency to evoke Evans blue extravasation in neonates than in adult fMV.[16] The present data suggest that temporal co-incidence of many glutamate receptors, low serine protease inhibition tone and weak ECM structure determine the structural background for immature vessel vulnerability to insult-evoked high glutamate. Identifying specific protease-substrate couples within the basal lamina and at EC basal pole during period of hemorrhage risk is the next step. However, the perspective of pharmacological vascular protection using antiprotease tools is a challenging issue since protease activities also displayed protective effects.[50]

Bleeding has become a major concern in neonatal intensive care units.[51,52] SEH/IVH/IPH shows preferential location in the germinal matrix. fMV extracted in neonatal mice were not obtained in the germinal matrix. Nevertheless, the identification of many maturation indices in the whole fMV opens new avenues of investigation towards the putative effectors of vessel disruption, in the at-risk area in collections of preterm brains. This present study opens new avenues for preventive action directed towards extracellular proteases. As proteases also participate in brain development, it is essential to identify specific activity for inhibition during the short window of vulnerability and thus prevent SEH/IVH/IPH without interfering with neonate development. Finally, the concept of ontogenetic replacement of microvessel proteins during the perinatal period must be taken into account in the care of immature patients. This implies that microvessel physiology in early postnatal brain, at least in part, involves different molecular actors than in adult physiology. Therefore, extreme caution is necessary: firstly to extrapolate function maturity in neonates using markers validated in adults; and secondly to apply in very young preterms therapeutic approaches based on adult function models.

Funding

The author(s) disclosed receipt of the following financial support for the research, authorship, and/or publication of this article: INSERM (Institut National de la Santé et de la

recherche Médicale), Région Haute-Normandie, EEC (European Economic Council) FEDER (Fonds Européen de Développement Régional (grants 33245 and 33267) and European Inter-Reg Program (Perene).

Acknowledgements

Mass spectrometry proteomic data were formatted using PRIDE convertor[53] and deposited in the ProteomeXchange Consortium[54] *via* the PRIDE partner repository with the dataset identifier PXD001718 and 10.6019/PXD0017188.

We are grateful to Nikki Sabourin-Gibbs, Rouen University Hospital, for writing assistance and review of the manuscript in English.

Declaration of conflicting interests

The author(s) declared no potential conflicts of interest with respect to the research, authorship, and/or publication of this article.

Authors' contributions

B.P.: Performed the work as doctoral fellow. J.H.: Mass spectrometry. Y.Z.: Microarrays. C.D.: Gene ontology analyses. M.H.: Biological material preparation. N.D.: Biological material preparation. A.O.: Protein handling, bioinformatics. T.L.: Transcriptomic platform supply. S.B.: Scientific support. B.G.: Scientific support. J-M.F.: Conception of transcriptome analyzes. S.M.: Initiation of the work, MS writing. P.C.: Conception of proteomic study, proteomic platform supply. P.L.: Initiation of the work, financing and direction of the study, MS writing.

Supplementary material

Supplementary material for this paper can be found at http://jcbfm.sagepub.com/content/by/supplemental-data

References

1. Volpe JJ. The encephalopathy of prematurity-brain injury and impaired brain development inextricably intertwined. *Semin Pediatr Neurol* 2009; 16: 167–178.
2. McQuillen PS and Ferriero DM. Selective vulnerability in the developing central nervous system. *Pediatr Neurol* 2004; 30: 227–235.
3. Ballabh P. Intraventricular hemorrhage in premature infants: mechanism of disease. *Pediatr Res* 2010; 67: 1–8.
4. Ballabh P, Braun A and Nedergaard M. Anatomic analysis of blood vessels in germinal matrix, cerebral cortex, and white matter in developing infants. *Pediatr Res* 2004; 56: 117–124.
5. Licht T, Dor-Wollman T, Ben-Zvi A, et al. Vessel maturation schedule determines vulnerability to neuronal injuries of prematurity. *J Clin Invest* 2015; 125: 1319–1328.
6. Takashima S, Itoh M and Oka A. A history of our understanding of cerebral vascular development and pathogenesis of perinatal brain damage over the past 30 years. *Semin Pediatr Neurol* 2009; 16: 226–236.
7. Volpe JJ. Intracranial hemorrhage: germinal matrix-intraventricular hemorrhage of the premature infant. In: Volpe JJ (ed.) *Neurology of the newborn*, 5th ed. Philadelphia: Saunders-Elsevier, 2008, pp.517–588.
8. Yang D, Baumann JM, Sun YY, et al. Overexpression of vascular endothelial growth factor in the germinal matrix induces neurovascular proteases and intraventricular hemorrhage. *Sci Transl Med* 2013; 5: 193ra190.
9. Braun A, Xu H, Hu F, et al. Paucity of pericytes in germinal matrix vasculature of premature infants. *J Neurosci* 2007; 27: 12012–12024.
10. Marin-Padilla M. The human brain intracerebral microvascular system: development and structure. *Front Neuroanat* 2012; 6: 38.
11. Fernandez-Lopez D, Faustino J, Daneman R, et al. Blood-brain barrier permeability is increased after acute adult stroke but not neonatal stroke in the rat. *J Neurosci* 2012; 32: 9588–9600.
12. Saunders NR, Daneman R, Dziegielewska KM, et al. Transporters of the blood-brain and blood-CSF interfaces in development and in the adult. *Mol Aspects Med* 2013; 34: 742–752.
13. Vannucci SJ and Simpson IA. Developmental switch in brain nutrient transporter expression in the rat. *Am J Physiol Endocrinol Metab* 2003; 285: E1127–E1134.
14. Adle-Biassette H, Olivier P, Verney C, et al. Cortical consequences of in vivo blockade of monocarboxylate transport during brain development in mice. *Pediatr Res* 2007; 61: 54–60.
15. Domoki F, Kis B, Gaspar T, et al. Cerebromicrovascular endothelial cells are resistant to L-glutamate. *Am J Physiol Regul Integr Comp Physiol* 2008; 295: R1099–R1108.
16. Henry VJ, Lecointre M, Laudenbach V, et al. High t-PA release by neonate brain microvascular endothelial cells under glutamate exposure affects neuronal fate. *Neurobiol Dis* 2013; 50: 201–208.
17. Legros H, Launay S, Roussel BD, et al. Newborn- and adult-derived brain microvascular endothelial cells show age-related differences in phenotype and glutamate-evoked protease release. *J Cereb Blood Flow Metab* 2009; 29: 1146–1158.
18. Wang X, Tsuji K, Lee SR, et al. Mechanisms of hemorrhagic transformation after tissue plasminogen activator reperfusion therapy for ischemic stroke. *Stroke* 2004; 35: 2726–2730.
19. Wang J and Tsirka SE. Contribution of extracellular proteolysis and microglia to intracerebral hemorrhage. *Neurocrit Care* 2005; 3: 77–85.
20. Xue M and Del Bigio MR. Injections of blood, thrombin, and plasminogen more severely damage neonatal mouse brain than mature mouse brain. *Brain Pathol* 2005; 15: 273–280.
21. Alles YC, Greggio S, Alles RM, et al. A novel preclinical rodent model of collagenase-induced germinal matrix/intraventricular hemorrhage. *Brain Res* 2010; 1356: 130–138.
22. Rosenberg GA, Mun-Bryce S, Wesley M, et al. Collagenase-induced intracerebral hemorrhage in rats. *Stroke* 1990; 21: 801–807.

23. Bauer AT, Burgers HF, Rabie T, et al. Matrix metalloproteinase-9 mediates hypoxia-induced vascular leakage in the brain via tight junction rearrangement. *J Cereb Blood Flow Metab* 2010; 30: 837–848.
24. Asahi M, Wang X, Mori T, et al. Effects of matrix metalloproteinase-9 gene knock-out on the proteolysis of blood-brain barrier and white matter components after cerebral ischemia. *J Neurosci* 2001; 21: 7724–7732.
25. Lekic T, Klebe D, McBride DW, et al. Protease-activated receptor 1 and 4 signal inhibition reduces preterm neonatal hemorrhagic brain injury. *Stroke* 2015; 46: 1710–1713.
26. Omouendze PL, Henry VJ, Porte B, et al. Hypoxia-ischemia or excitotoxin-induced tissue plasminogen activator-dependent gelatinase activation in mice neonate brain microvessels. *Plos One* 2013; 8: e71263.
27. Suzuki Y, Nagai N, Umemura K, et al. Stromelysin-1 (MMP-3) is critical for intracranial bleeding after t-PA treatment of stroke in mice. *J Thromb Haemost* 2007; 5: 1732–1739.
28. Yepes M, Sandkvist M, Moore EG, et al. Tissue-type plasminogen activator induces opening of the blood-brain barrier via the LDL receptor-related protein. *J Clin Invest* 2003; 112: 1533–1540.
29. Leroux P, Omouendze PL, Roy V, et al. Age dependent neonatal intracerebral hemorrhage in plasminogen activator inhibitor-1 (PAI-1) knock-out mice. *J Neuropathol Exp Neurol* 2014; 73: 387–402.
30. Vezain M, Saugier-Veber P, Melki J, et al. A sensitive assay for measuring SMN mRNA levels in peripheral blood and in muscle samples of patients affected with spinal muscular atrophy. *Eur J Hum Genet* 2007; 15: 1054–1062.
31. Huang da W, Sherman BT and Lempicki RA. Bioinformatics enrichment tools: paths toward the comprehensive functional analysis of large gene lists. *Nucleic Acids Res* 2009; 37: 1–13.
32. Huang da W, Sherman BT and Lempicki RA. Systematic and integrative analysis of large gene lists using DAVID bioinformatics resources. *Nat Protoc* 2009; 4: 44–57.
33. Kanehisa M and Goto S. KEGG: Kyoto encyclopedia of genes and genomes. *Nucleic Acids Res* 2000; 28: 27–30.
34. Kanehisa M, Goto S, Sato Y, et al. Data, information, knowledge and principle: back to metabolism in KEGG. *Nucleic Acids Res* 2014; 42: D199–D205.
35. Chun HB, Scott M, Niessen S, et al. The proteome of mouse brain microvessel membranes and basal lamina. *J Cereb Blood Flow Metab* 2011; 31: 2267–2281.
36. Ek CJ, Dziegielewska KM, Habgood MD, et al. Barriers in the developing brain and neurotoxicology. *Neurotoxicology* 2012; 33: 586–604.
37. Gentil BJ, Benaud C, Delphin C, et al. Specific AHNAK expression in brain endothelial cells with barrier properties. *J Cell Physiol* 2005; 203: 362–371.
38. Kuperman AA, Kenet G, Papadakis E, et al. Intraventricular hemorrhage in preterm infants: coagulation perspectives. *Semin Thromb Hemost* 2011; 37: 730–736.
39. Ballabh P, Xu H, Hu F, et al. Angiogenic inhibition reduces germinal matrix hemorrhage. *Nat Med* 2007; 13: 477–485.
40. Minagar A, Alexander JS, Kelley RE, et al. Proteomic analysis of human cerebral endothelial cells activated by glutamate/MK-801: significance in ischemic stroke injury. *J Mol Neurosci* 2009; 38: 182–192.
41. Pottiez G, Deracinois B, Duban-Deweer S, et al. A large-scale electrophoresis- and chromatography-based determination of gene expression profiles in bovine brain capillary endothelial cells after the re-induction of blood-brain barrier properties. *Proteome Sci* 2010; 8: 57.
42. Uchida Y, Ohtsuki S, Katsukura Y, et al. Quantitative targeted absolute proteomics of human blood-brain barrier transporters and receptors. *J Neurochem* 2011; 117: 333–345.
43. Sashindranath M, Sales E, Daglas M, et al. The tissue-type plasminogen activator-plasminogen activator inhibitor 1 complex promotes neurovascular injury in brain trauma: evidence from mice and humans. *Brain* 2012; 135: 3251–3264.
44. Montaner J, Molina CA, Monasterio J, et al. Matrix metalloproteinase-9 pretreatment level predicts intracranial hemorrhagic complications after thrombolysis in human stroke. *Circulation* 2003; 107: 598–603.
45. Suzuki Y, Nagai N, Yamakawa K, et al. Tissue-type plasminogen activator (t-PA) induces stromelysin-1 (MMP-3) in endothelial cells through activation of lipoprotein receptor-related protein. *Blood* 2009; 114: 3352–3358.
46. de Vries LS, Koopman C, Groenendaal F, et al. COL4A1 mutation in two preterm siblings with antenatal onset of parenchymal hemorrhage. *Ann Neurol* 2009; 65: 12–18.
47. Gould DB, Phalan FC, Breedveld GJ, et al. Mutations in Col4a1 cause perinatal cerebral hemorrhage and porencephaly. *Science* 2005; 308: 1167–1171.
48. McCarty JH, Monahan-Earley RA, Brown LF, et al. Defective associations between blood vessels and brain parenchyma lead to cerebral hemorrhage in mice lacking alpha V integrins. *Mol Cell Biol* 2002; 22: 7667–7677.
49. Lecointre M, Hauchecorne M, Chaussivert A, et al. The efficiency of glutamate uptake differs between neonatal and adult cortical microvascular endothelial cells. *J Cereb Blood Flow Metab* 2014; 34: 764–767.
50. Cheng T, Petraglia AL, Li Z, et al. Activated protein C inhibits tissue plasminogen activator-induced brain hemorrhage. *Nat Med* 2006; 12: 1278–1285.
51. Marret S, Marchand-Martin L, Picaud JC, et al. Brain injury in very preterm children and neurosensory and cognitive disabilities during childhood: the EPIPAGE cohort study. *PLos One* 2013; 8: e62683.
52. Ancel PY, Goffinet F, Group E-W, et al. Survival and morbidity of preterm children born at 22 through 34 weeks' gestation in France in 2011: results of the EPIPAGE-2 cohort study. *JAMA Pediatr* 2015; 169: 230–238.

53. Cote RG, Griss J, Dianes JA, et al. The PRoteomics IDEntification (PRIDE) Converter 2 framework: an improved suite of tools to facilitate data submission to the PRIDE database and the ProteomeXchange consortium. *Mol Cell Proteomics* 2012; 11: 1682–1689.

54. Vizcaino JA, Deutsch EW, Wang R, et al. ProteomeXchange provides globally coordinated proteomics data submission and dissemination. *Nat Biotechnol* 2014; 32: 223–226.
