# Supplementary material for: Proteomic and transcriptomic study of brain microvessels in neonatal and adult mice
Source: PLoS One. 2017 Jan 31;12(1):e0171048. doi: 10.1371/journal.pone.0171048 (PMC5283732; doi:10.1371/journal.pone.0171048)
Supplement: S3 File — (PDF) [file pone.0171048.s003.pdf]

Liberté • Égalité • Fraternité
RÉPUBLIQUE FRANÇAISE

MINISTERE DE L'ÉDUCATION NATIONALE,
DE L'ENSEIGNEMENTSUPERIEUR ET DE LA RECHERCHE

Direction générale
de la recherche
et de l'innovation

Service de la performance,
du financement et de la
contractualisation avec les
organismes de recherche

Département de la culture
scientifique et des relations
avec la société

Expérimentation animale -
Autorisation de projet

Affaire suivie par
Virginie Vallet-Erdtmann
Chargée de mission

Téléphone
01 55 55 99 55
Fax
01 55 55 99 59
Mél.
Virginie.vallet-erdtmann
@recherche.gouv.fr

1 rue Descartes
75231 Paris Cedex 05

Paris, le 13 OCT. 2014

Objet : Notification d'autorisation de projet utilisant des animaux à des fins scientifiques

Monsieur,

En application des dispositions du code rural et de la pêche maritime, notamment ses articles R. 214-87 à R.214-126, le projet :

- référencé sous le numéro *01680.02,*
- ayant pour titre : « *Recherche d'agents thérapeutiques à visée neuroprotectrice vis-à-vis des lésions cérébrales périnatales. Modélisation de plusieurs types de lésions (ischémiques, hémorragiques ou toxiques) chez la Souris.* »,
- déposé par l'Établissement Utilisateur : *UFR Médecine-Pharmacie,* numéro d'agrément *B7645005* dont le responsable est *Monsieur Pierre Fréger,*
- et dont les responsables de la mise en œuvre générale du projet et de sa conformité à l'autorisation sont *Monsieur Bruno Gonzalez, Madame Sylvie Jegou, Monsieur Philippe Leroux, Madame Carine Cleren, Madame Isabelle Leroux-Nicollet, Madame Nathalie Dourmap, Madame Carole Brasse-Lagnel, Madame Michèle Hauchecorne,*

est autorisé.

L'autorisation de projet est accordée pour une durée de *5 ans* à partir du 13 OCT. 2014

Le projet est autorisé sous réserve de l'obtention formelle de l'agrément par l'Établissement Utilisateur.

Le projet précité a été évalué sur le plan éthique par le Comité d'éthique en expérimentation animale n°*54* et a reçu un avis *favorable*.

Toutefois, le comité d'éthique a proposé le reclassement des procédures expérimentales *n°1, n°2, n°3 et n°4* du projet, de la classe « légère » à la classe « *modérée* ».

Ce projet ne fera pas l'objet, à l'issue de sa réalisation, d'une appréciation rétrospective.

Pour la ministre et par délégation
le chef du service de la performance, du
financement et de la contractualisation avec
les organismes de recherche

Éric BERNET

Monsieur Pierre Fréger
pierre.freger@univ-rouen.fr
